# Education, Dietary Intakes and Exercise*

**DOI:** 10.1111/obes.12463

**Published:** 2021-09-16

**Authors:** Stephanie Von Hinke

**Affiliations:** † School of Economics, University of Bristol, 12 Priory Road, Bristol BS8 1TU, UK; ‡ Erasmus School of Economics, Erasmus University Rotterdam, Rotterdam, The Netherlands; § Institute for Fiscal Studies, London, UK

**Keywords:** I12, I21, I28

## Abstract

This paper examines the relationship between education and health behaviours, focusing on potential offsetting responses between calories *in* (i.e. dietary intakes) and calories *out* (i.e. physical activity). It exploits the 1972 British compulsory schooling law that raised the minimum school leaving age from 15 to 16 to estimate the effects of education on diet and exercise around middle age. Using a regression discontinuity design, the findings suggest that the reform led to a *worsening* of the quality of the diet, with increases in total calories, fats and animal proteins. However, I find that these changes are partially offset by a discontinuous increase in physical activity. Back-of-the-envelope calculations suggest little effect on the balance of calories. As such, the findings show that focusing on the two components of energy balance provides additional information that is concealed in analyses that only use a measure of obesity.

## I Introduction

There is a strong social gradient in health and disease, with lower socio-economic classes experiencing increased morbidity. One of the main causes of this are poor dietary choices (WHO, 2002 Marmot, 2005). Governments across the world are trying to encourage individuals to make healthier choices, through channels such as information provision (e.g. the five-a-day campaign (Capacci and Mazzochi, 2011), food labelling (Fichera and von Hinke, 2020)) and fiscal measures (e.g. taxes (Fletcher, 2010), targeted benefits (Griffith, von Hinke and Smith, 2018)).

Given the social gradient in nutritional choices, one question is whether we can improve individuals’ diets by increasing their socio-economic status, such as education. Indeed, the model for the demand for health (Grossman, 1972) suggests that education affects health *directly* via the accumulation of knowledge and improved cognitive functioning. For example, education can change individuals’ allocative efficiency, affecting the allocation of health inputs such as dietary choices. Education may also *indirectly* affect health inputs by increasing earnings, which can in turn affect nutritional choices. For example, higher wages may increase the affordability of health-improving foods. However, higher wages also increase the opportunity cost of time, potentially leading to individuals increasing their consumption of (time-saving) ready meals, which tend to be less healthy.^1^

This paper examines the relationship between education and the nutritional composition of the diet, with two main contributions. First, with little evidence on the causal effect of education on dietary choices, this paper fills this gap in the literature. Because of the importance of dietary choices in determining individuals’ body weight, one could indirectly explore whether education affects diets by investigating its effect on obesity. Indeed, various studies have taken this approach, but they tend to find no consistent evidence of such a relationship (for a recent review, see Galama, Lleras-Muney and van Kippersluis (2018)). However, as obesity is a function of calories *in* as well as *out*, using obesity as the main outcome may conceal more disaggregated effects coming from the two separate components.

The second contribution, therefore, comes from being able to shed light on the potential offsetting responses between calories in and calories out. Hence, I investigate the causal effect of education on dietary choices as well as individuals’ physical activity. I exploit national changes to the UK minimum school leaving age introduced in 1972 as exogenous variation in years of schooling in an IV setup. More specifically, the UK government increased the age at which individuals were legally allowed to leave school from 15 to 16 on 1 September 1972. This meant that everyone born prior to 1 September 1957 was allowed to leave school when they turned 15, whereas those born on or after 1 September 1957 had to stay in school until they turned 16. The 1972 reform has been exploited for analyses in other contexts, including those estimating the effect of education on, for example, wages (see e.g. Harmon and Walker, 1995; Oreopoulos, 2006), health and mortality (see e.g. van Kippersluis, O’Donnell and van Doorslaer, 2009; Davies *et al*., 2018; Janke *et al*., 2018; Clark and Royer, 2013), family formation (Hener and Wilson, 2015; Geruso and Royer, 2018), teenage motherhood (Wilson, 2014) and decision making (see e.g. Banks, Carvalho and Perez-Arce, 2018).^2^

Consistent with these studies, I find that the 1972 schooling reform increased years of education. The results for dietary choices, however, suggest that additional education *reduces* the nutritional quality of the diet. More specifically, I find that education increases purchases of total calories, fats and animal proteins. These findings are robust to a range of alternative model specifications, and I replicate these results using a different dataset to look at nutrient *consumption* as opposed to nutrient *purchases*. The results show similar patterns, although with a substantially smaller sample size, they are not significantly different from zero.

Next, I explore potential mechanisms of this effect, highlighting two channels in particular. First, I explore the role of income in explaining the increase in nutrient purchases, but I find no evidence of income driving this effect. Second, I investigate the role of alcohol and smoking. The analysis shows some suggestive evidence that education increases alcohol (but not tobacco) spending. Since alcohol and food are often considered complements, this suggests that the increased alcohol spending among the higher educated may explain some of the increase in nutrient purchases among this group.

Finally, I investigate the effect of education on calories *out*, exploring whether the worsening of the diet is mitigated by changes in levels of (sports-related) physical activity. Using the same empirical strategy, I find that additional education *increases* physical activity, suggesting that the reduction in the dietary quality is partially offset by increased physical exercise. Furthermore, I find no significant change in occupation-related strenuousness. A back-of-the-envelope calculation suggests that the change in calories *in* is likely to be only slightly larger compared to the change in calories *out*. Although these are rather crude estimates due to data limitations and measurement error, this suggests that there is little effect on the balance of calories. There are at least two explanations for this finding. First, individuals may choose to exercise more to allow them to consume more unhealthy foods (i.e. a licensing effect), and this may differ by education. Second, higher educated individuals may be more aware of the importance and benefits of physical activity (i.e. a knowledge effect), and doing more exercise requires more foods to sustain this higher level of physical activity. Although I am unable to identify the exact mechanism (e.g. a licensing vs. knowledge effect), the analysis highlights the importance of separately exploring the effects on calories in vs. calories out, providing additional information that is concealed in the analyses that only use measures of obesity.

With that, this paper speaks to a large literature that explores the effects of education on health. Much of this literature exploits compulsory schooling reforms to estimate the effects, with some mixed results. While many studies suggest education does not have strong causal effects on morbidity or mortality (see e.g. Albouy and Lequien, 2009; Clark and Royer, 2013; Jurges, Kruk and Reinhold, 2013, the latter possibly driven by a lack of power), some do find evidence of a negative effect (e.g. Lleras-Muney, 2005; van Kippersluis *et al*., 2009; Davies *et al*., 2018), or find such evidence for a selection of health outcomes (e.g. Janke *et al*., 2018). Other effects of compulsory schooling that have been found in the literature are improvements in self-assessed health (Silles, 2009), reductions in long-term illness (Kemptner, Jurges and Reinhold, 2011), worse mental health (potentially because it forces low achieving teenagers to remain in an academic environment; Avendano, de Coulon and Nafilyan, 2017), but no changes in health knowledge (Johnston *et al*., 2015), smoking or obesity (see e.g. Galama *et al*., 2018). To the best of my knowledge, this study is the first to include a detailed investigation of the effect of education on the two main subcomponents that determine obesity, calories in and calories out, and with that show their substantial offsetting effects.

A handful of studies have explored the specific effects on nutritional choices. For example, Li and Powdthavee (2015) estimate the effect of education on the likelihood of complying with certain dietary guidelines (e.g. regular consumption of fruit and vegetables). They find that education increases compliance, with some improvements in self-assessed health. Atella and Kopinska (2014) and Fletcher (2015) estimate the effect of education on total calorie intakes, but not nutrients, while Barcellos, Carvalho and Turley (2017) focus on macronutrients, as measured from respondents’ reported consumption of 21 specific food groups. Using a similar research design that exploits compulsory schooling laws in the United States, Fletcher (2015) finds that an additional year of schooling decreases daily caloric intake by 87 calories and increases vitamin use (another dimension of dietary quality) by 31 percentage points, although these are not statistically significantly different from zero. The findings in this paper suggest the opposite for the United Kingdom: a drop in dietary quality, albeit offset by an increase in physical activity.

The paper also contributes to the literature that examines individuals’ compensatory responses. The existing evidence on such effects within economics includes, for example, evidence on how individuals offset additional calories consumed in restaurants by cutting back on caloric intakes at other times (Anderson and Matsa, 2011); how parental investments in children may compensate for adverse (early life) shocks (see e.g. Almond and Currie, 2011); or how tobacco tax hikes lead smokers to compensate by extracting more nicotine per cigarette (see e.g. Adda and Cornaglia, 2006). The literature that specifically explores interactions between energy intakes and expenditures discusses homeostatic body weight regulation, which implies that body weight returns to base-line after any impositions, due to (innate) compensatory adjustments in energy balance (see e.g. Epstein and Wing, 1980; King *et al*., 2007). Similarly, the economics literature emphasizes the importance of both channels to understand recent changes in obesity over time (Cutler, Glaeser and Shapiro, 2003; Griffith, Lluberas and Lührmann, 2016). This paper confirms this compensatory channel and shows that the reduction in dietary quality due to additional education is offset by increased physical exercise, or vice versa, that the increased physical activity due to additional education is offset by increased food intake. Taken together, there is little effect on the balance of calories consumed.

The rest of the paper is structured as follows: the next section explains the compulsory schooling reform. Section III describes the data, followed by the empirical approach in section IV. Section V presents the results, and section VI concludes.

## II The compulsory schooling reform

**Figure 1:**
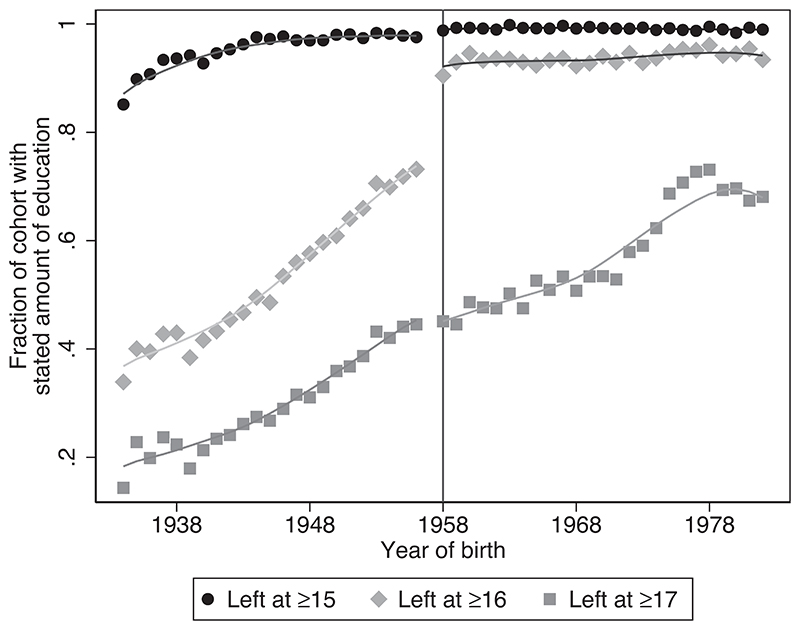
Years of full-time education by birth cohort Notes The sample includes all individuals with a year of birth between 1934 and 1982 from the Living Cost and Food Survey, 2003–15. The vertical line represents the cut-off indicating the first cohort subject to the new compulsory schooling law

On 1 September 1972, the United Kingdom increased the age at which individuals were legally allowed to leave school from 15 to 16. This meant that everyone born prior to 1 September 1957 was allowed to leave school when they turned 15, whereas those born on or after 1 September 1957 had to stay in school until they turned 16. Figure 1 illustrates the impact of this reform on the proportion of individuals leaving school at age ≥15, ≥16 and ≥17 years old. The horizontal axis shows the year of birth, with the vertical bar denoting 1958: the first full birth cohort subject to the 1972 compulsory schooling reform. The lines are obtained from locally weighted regressions, estimated separately for cohorts born before and after 1957. It shows a clear discontinuity in the probability of leaving school at age 16 or older for the cohorts born after this cut-point, with no large changes in the proportion of individuals leaving education at age 15 or 17.

## III Data

### The Living Cost and Food Survey

The main dataset I use is the 2003 to 2015 Living Cost and Food Survey (LCFS; known as the Expenditure and Food Survey (EFS) between 2001 and 2007), which provides information about spending patterns, cost of living, food purchases and nutrition. The data are repeated cross-sections of around 6,000 randomly selected households each year in the United Kingdom. Individuals are asked to keep a diary, where they record all daily expenditures for a period of 14 days, including foods purchased for home as well as out. Hence, the analysis below concerns food *purchases* as opposed to food *consumption*. The analysis focuses on the main shopper in the household, defined as the person with the highest food spending, who is assumed to be the main meal planner.

The advantages of these data are that they include a very detailed breakdown of households’ shopping baskets, and of the nutrients that make up the shopping basket, allowing me to explore the effects of education on individual nutrients, such as unsaturated vs. saturated fats, and animal vs. plant protein.^3^ Other data do not record nutritional information with this level of detail. Furthermore, surveys of food *consumption* that do include detailed nutritional information, such as the UK National Diet and Nutrition Survey (NDNS), generally contain much smaller sample sizes. Nevertheless, I will use these data to explore the robustness of my analyses below. As with all data, however, there are some disadvantages. One limitation is that the actual nutrient purchases are only measured at the level of the household. Hence, in the analyses, I relate the main shopper’s level of education to *household* nutrient purchases. I show below, however, that the main shopper is responsible for the majority (83%) of all grocery spending.

Another limitation is that, similar to, for example, Oreopoulos (2006), I only observe year of birth, rather than month of birth. Hence, as the reform differentially affected those born before and after 1 September 1957, this does not allow me to take that into account in the analyses, meaning I cannot assign treatment status at the month level. To avoid erroneously assigning individuals to being born before or after the cut-off, I drop the year 1957 from the analyses and compare those born up to 1956 to those born in 1958 onwards. This is also known as a ‘donut’ regression discontinuity (Barreca *et al*., 2011). The advantage is that one does not need to worry about measurement error due to partially unobserved (or misreporting) of the running variable. However, a disadvantage is that the estimates require more extrapolation due to the omission of data immediately around the threshold. Furthermore, it means that treatment and control groups are not precisely assigned at the threshold and it does not allow me to capture local trends immediately around the cut-off using, for example, a local linear specification (see e.g. Clark and Royer, 2013).

To measure the quantity of nutrients purchased by each household, I use the conversion factors provided in the data. These measure the amount of each nutrient per kg of a highly disaggregated group of foods. Hence, multiplying the quantity purchased with its conversion factor gives the total amount of nutrients purchased over the 14 day period. I then divide this by 14 to obtain daily nutrient purchases. Finally, I create a ‘nutrient equivalence scale’ and use this to obtain the *average daily nutrient purchases per person*.^4^

I am interested in the following key nutrients that have been identified as important for health and development: energy (kcal), carbohydrates (including sugar and fibre), fats and proteins. In addition, in line with current dietary guidelines, I will separately explore fats and saturated fats, and distinguish between total sugars and non-milk extrinsic sugars (NMES).^5^ I will also separate out animal and plant protein. In addition to examining households’ purchases of specific nutrients, I combine the nutritional information to obtain a score that indicates the ‘healthiness’ of the household diet. For this, I use the Nutritional Profile Model, developed by the UK Food Standards Agency (Rayner *et al*, 2009) and used by the UK Media regulator Ofcom. In short, this model attaches a score depending on the amount of each of the nutrients it contains per 100g. The Nutritional Profile Score measures the nutritional quality of the shopping basket, where higher scores indicate less healthy baskets. For more information on the Nutritional Profile Model, see Appendix A.

To obtain the estimation sample, I drop households where the woman is pregnant, as this may affect the quantity and types of nutrients purchased. This leaves me with 74,080 households observed between 2003 and 2015. Next, I restrict the sample to the 1934–82 birth cohorts and exclude 1957; that is, 24 cohorts born prior to, and after the reform, leading to 60,764 households.^6^ I check the robustness of my results by reducing this bandwidth below. Finally, I drop 5,178 households where the school leaving age is unknown, and drop five households who do not spend any money on food in the relevant 14-day period in which they were asked to keep a diary, leading to an estimation sample of 55,581 households.

### Descriptive statistics

Table 1 presents the descriptive statistics for the final sample of households, where any individual-level characteristics refer to the main shopper. This shows that the main shopper is male in 34% of households, with an average age of 48. Across all households, the main shopper is responsible for 83% of all grocery spending.^7^ Over half of the sample consists of households with two adult members, 15% have one child, and 20% have two or more children.

Figure C.1 and Figure C.2 in Appendix C show the distributions of the different nutrients, where all nutrients have been equivalized using the ‘nutrient equivalence scale’ discussed above, showing a near-normal distribution for the overall Nutritional Profile Score, and smooth densities for all individual nutrients.

## IV Empirical methodology

I exploit the 1972 UK compulsory schooling reform as an instrument for education, using a regression discontinuity (RD) design. As not all individuals comply with the reform, I specify a fuzzy RD approach, estimated by two-stage least squares. This approach exploits the fact that individuals’ years of schooling is a non-continuous function of the forcing variable (i.e. year of birth), with a discontinuity at the threshold (see e.g. Imbens and Lemieux, 2008; Lee and Lemieux, 2010). In other words, only an individual’s year of birth determines whether or not the person is exposed to the schooling reform.

In the first stage, I specify a dummy *D_i_* that equals 1 if the individual *i* is exposed to the reform (i.e. born in or after 1958) and zero otherwise as an instrument for the measure of education. I estimate the effects of this reform on a binary variable indicating whether the main shopper left school at age 16 or older, denoted by *E_i_*:

$$E_{i}=\alpha_{0}+\alpha_{1}D_{i}+f(YOB_{i})+\gamma X_{i}+\nu_{i},$$

where *f*(*YOB_i_*) incorporates a nonlinear (quadratic) function in the year of birth of the main shopper relative to the cut-off, as well as that interacted with the treatment dummy, allowing for differential nonlinear trends for cohorts born before and after the threshold.^8^. The vector **X_i_** captures a set of background characteristics, including a quadratic in age, year and month dummies, gender, marital status, variables indicating the number of adults in the household, the number of children aged 0, aged 1, . . ., aged 17, and region dummies. However, as these covariates should be uncorrelated to being born before or after the cut-off, its inclusion should not result in large changes in the estimate of the interest. Figure 1 graphically shows the extent to which the reform changed levels of education for the affected cohorts. The regression will quantify this shift and show the robustness to the inclusion of additional trends and covariates.

In the second stage, I examine the causal effect of education on nutritional purchases, using the dummy for being exposed to the reform (*D_i_*) as the instrument for education (*E_i_*) in a two-stage least squares (2SLS) regression.^9^ The equation is given by:

(1) $$Y_{i}^{j}=\beta_{0}+\beta_{1}E_{i}+f(YOB_{i})+\zeta X_{i}+\varepsilon_{i},$$

where $Y_{i}^{j}$ denotes the average daily nutrient *j* purchased by individual *i*, with *i* = 1, . . ., *N* and *j* = 1, . . ., *J*, and *E_i_* denotes the measure of education that is instrumented by the dummy *D_i_*. The function *f(YOB_i_)* again denotes the flexible function in the year of birth relative to the cut-off, **X_i_** is a vector of covariates, and the error term is given by *ε_i_*. All analyses are clustered at the birth cohort level. For all analyses, I use the weights provided by the data to adjust for non-response and ensure representativeness of the population.

It is worth highlighting some potential issues when exploiting the increase in the minimum school leaving age, which may affect the estimation and interpretation of the parameters. First, this policy may have led to complex general equilibrium effects, some of which may be working through the marriage market (see e.g. Akresh, Halim and Kleemans, 2018; Anderberg *et al*., 2019). For example, the rise in the minimum school leaving age may have resulted in more marriages *within* cohorts due to assortative mating, where the reform has increased education for both partners. In the analysis above, I only account for the main shopper’s level of education, ignoring the spouse’s education that may have similarly increased due to the reform, since spouse’s education is missing for a large number of observations. This in turn may lead to an upward bias in the estimates. I present the analysis that accounts for spouse’s education in the robustness section in Appendix B. Similarly, the rise in the minimum school leaving age may have affected marital sorting patterns. Indeed, Anderberg *et al*. (2019) find that the reform led to increases in never-married rates among the least educationally qualified in society. This differential sorting may independently affect individuals’ decisions, including their nutritional choices.

Another potential issues is that, due to the higher wages driven by the increased education, higher educated individuals may decide to purchase *more expensive* or *higher quality* foods. As the analysis does not explore individuals’ food expenditures itself, but instead focuses on total nutrients purchased, this is not an issue *per se*. However, it does affect the interpretation of the estimates. In particular, as discussed in section III, the dependent variable is obtained by multiplying the conversion factors for each highly disaggregated group of foods with the quantity purchased of that food group. The conversion factors record the *average* nutrients per kg of each food group. This implies that any systematic differences in the nutritional composition of *higher* compared to *lower* quality foods *within a food group* may bias the estimates. For example, if more expensive foods within a particular food group are nutritionally better (e.g. less sugar) and these are systematically more likely to be purchased by higher educated individuals, this would overestimate the parameter of interest.

Unfortunately, as the data provide nutrient conversion factors that differ only *across* food groups, but not *within*, it is not possible to explore the importance of this in more detail. However, the data include over 500 food groups in total, and although there is nutritional variation within these, they are designed to capture similar types of foods within each category (e.g. there are separate categories for oatmeal, muesli, high fibre breakfast cereals, sweetened breakfast cereals, other breakfast cereals, cereal bars and cereal snacks). Other categories also distinguish between price/quality of foods (e.g. white bread premium, white bread soft grain or white bread standard; less expensive beef steak, and more expensive beef steak), suggesting that the disaggregated food groups capture the majority of the variation in nutritional composition. Nevertheless, there remains some variation in the nutritional composition of foods *within* food groups (Griffith and O’Connell, 2009), suggesting that the estimates are likely to be an upper bound of the ‘true’ effect, which should be taken into account when interpreting the results.

Furthermore, because the parameters are estimated using IV, the estimates pick up a local average treatment effect (LATE). In other words, they capture the effect of staying in school until age 16 on nutritional choices for those who would have left at age 15 in the absence of the policy. This is a specific group of individuals, meaning that the estimates do not necessarily generalize to the population. Nevertheless, it is an interesting group for at least two reasons. First, as I show in section V below, the reform affects a relatively large part of the population. And second, individuals affected by the policy are currently in their 60s, who have obesity rates that are among the highest in the United Kingdom (Baker, 2018).

## V Results

### Nutritional choices

I start by discussing the findings from the naïve OLS regressions, followed by the IV analyses. Table 2 shows the former, regressing the Nutrient Profile Score (column 1) or the average amount of nutrients purchased per day (columns 2–13) on a binary indicator for whether the individual left full-time education at age ≥16 and the other covariates discussed above. The results show that the higher educated, on average, have healthier diets: their Nutrient Profile Score is 0.25 units lower than the lower educated. With a standard deviation of the Nutrient Profile Score of 1.9 for cohorts born just before the cutoff (not shown here), this change is similar to approximately 13% of a standard deviation. Furthermore, the estimates suggest that the higher educated purchase significantly fewer calories, carbohydrates (including NMES), starch, fats (including saturated fats), sodium and animal protein, while they purchase more fibre and vegetable protein. For example, households where the main shopper left full-time education at age ≥16 purchase 51 fewer calories per person per day. With average purchases of 2610 calories per person for cohorts born just before the cut-off, this is approximately 2%. For the other nutrients, the differences between nutrient purchases of those who left education at age ≥16 and those who left before are all in the range of 1–6%, apart from proteins, where it is less than 1%. However, the latter is driven by a positive correlation with vegetable protein and a negative correlation with animal protein.

Table 3 quantifies the discontinuity in the probability of leaving school at age 16 from Figure 1, measuring the difference in the probability of leaving school at age ≥16 for those born before vs. in or after 1958. This shows that those born in 1958 onwards are 12 percentage points more likely to stay in school until age 16. With a pre-reform mean of 73%, this suggests that the reform led to a 16% increase in staying in school until age 16. The first stage F-statistic of the preferred specification (column (4)), is 53. Recent work by Lee *et al*. (2020) shows that the usual critical value of 1.96 for a test at five per cent significance level requires the first stage F-statistic to be greater than 104.7. For F-statistics below 104.7, the IV confidence intervals are wider. They show that the critical value for an F-statistic of 53.5 is 2.13. In addition to the standard IV results, I therefore also report the adjusted confidence intervals for all IV estimates, defined as $[{\hat{\beta }}_{1}^{IV}\pm 2.13\times SE({\hat{\beta }}_{1}^{IV})].$ I use the ‘standard’ 95% confidence intervals for all OLS and reduced form estimates.

Table 4 shows the results that instrument the measure of education using the dummy for being born in or after 1958.^10^ Using a standard 95% confidence interval (not shown here) would suggest that additional education *increases* purchases of calories, total sugars, NMES, starch, fats, saturated fats and animal proteins. Using the ‘*tF*’ procedure from Lee *et al*. (2020), however, only calories, fats and animal proteins remain significantly different from zero at the 5% level. This shows there is a reduction in the quality of the overall diet, captured by a 0.5 unit increase in the Nutrient Profile Score, although the latter is not significantly different from zero.

Looking at the magnitude of the effects, the estimates suggest that leaving school at 16 increases total calories by 619 per person per day, compared to those leaving school earlier. This is a large effect. There are at least three potential statistical explanations for the magnitude of this effect. First, as discussed above, if the higher educated are systematically more likely to purchase nutritionally better foods *within* a food group compared to the lower educated, perhaps because of their higher disposable income, this will lead to the parameter of interest being over-estimated, and hence, this should be interpreted as an upper bound of the ‘true’ effect. Second, if the reform led to an increased number of marriages *within* cohorts (e.g. due to assortative mating), the analysis may partially capture spouse’s level of education, leading to an upward bias. Due to substantial missing data on spouse’s education (33% of our estimation sample), I do not include this in the analysis. As expected, this shows smaller estimates with larger standard errors, but they remain substantial. Third, as the estimates represent a LATE, the coefficients can be interpreted as the causal effect of staying in school until age 16 among those would have left at age 15 had the policy not been introduced. Appendix B shows that the findings are robust to a large set of sensitivity analyses (including controlling for a proxy for income, restricting the sample to ages with common support in treated and control groups, reducing the bandwidth, and controlling for spouse’s level of education), although the magnitudes of the estimates for sugar and NMES are less robust.

Nevertheless, given the magnitude of the effects, it is important to consider potential mechanisms that may be driving this. There are at least two mechanisms to highlight. First, the additional income that is associated with staying in school may be used to purchase more foods, thereby increasing the total amount of energy and nutrients purchased among those who stayed in school because of the reform. Second, the higher educated may be more or less likely to drink alcohol and smoke. Both of these health behaviours have been found to affect individuals’ nutritional choices, and may explain the estimates found above. I will explore each of these in turn. Following that, I will highlight potential behavioural changes that may offset the negative health consequences of increased food intake.

### Potential mechanisms

One of the mechanisms that may explain the increased nutritional purchases is income. It is well known that the UK schooling reform led to higher wages for those affected (see e.g. Harmon and Walker, 1995; Oreopoulos, 2006). The increase in the amount of nutrients purchased may therefore be driven by increased income for these individuals. That is, if food is a normal good, individuals with higher disposable income may eat more, increasing the total amount of energy and nutrients purchased (unless they have already reached their saturation point). I do not control for income in the main analysis for two reasons. First, being exposed to the schooling reform causally affects earnings, so controlling for such ‘downstream’ variables may lead to biased estimates of the causal effect of interest. Second, the data do not include any measure of income. However, to explore the sensitivity of the estimates, I follow the literature and rerun the analyses on nutritional choices controlling for total household spending as a proxy for income (as in e.g. Griffith *et al*., 2018). The results are presented in Table 5, where Panel A presents the estimates that control for a linear specification of total spending, and Panel B controls for a quadratic in total spending. Both show no substantial changes in the IV estimates, with some additional evidence of an increase in purchases of non-milk extrinsic sugars, suggesting that income does not play a major role in explaining the increase in nutritional choices.

Other potential mechanisms that may be driving some of the effect of education on nutritional choices are alcohol consumption and smoking. Indeed, alcohol is often considered a complement to food, whereas smoking may be a substitute.^11^ This may mean that increased alcohol consumption among higher educated may be accompanied by increased food intake. Similarly, as smoking is more common among the lower educated, this may be one reason why they have reduced food purchases. Table 6 shows the IV estimates of effect of education on different measures of smoking and drinking, using the same specification as above.^12^ Columns (1) and (2) show the effect of education on a dummy indicating whether the household spent any money on alcohol, and the expenditures (in £) respectively. Column (3) and (4) show the same for tobacco. The findings indicate that although the IV estimates on alcohol spending are positive and relatively large, the adjusted confidence intervals using the *‘tF’* procedure renders them insignificantly different from zero at the 5% level. They are marginally significant at the 10% level, providing suggestive evidence that staying in school for longer increases household spending on alcohol. More specifically, staying in school until age 16 increases the probability of positive spending on alcohol by 24 percentage points, or £ 1.63 per week, on average. Since alcohol and food are considered complements, this suggests that the increased alcohol spending among the higher educated may explain some of the increase in nutrient purchases among this group. I find no significant effects for tobacco spending.

### Potential offsetting effects

Since the literature that looks at the health effects of additional education either finds no changes or improvements in (physical) health, finding that education reduces the nutritional quality of the diet is somewhat counter-intuitive. Indeed, if education negatively affects morbidity, we would expect to find a positive effect of education on dietary quality. Similarly, if education does not affect morbidity, we would expect to find no effects on dietary quality, unless any dietary changes are offset in others ways. I here explore the latter, estimating whether additional education affects other behaviours, potentially mitigating any effects of the reduction in nutritional quality.

More specifically, a reduction in dietary quality does not necessarily affect health if it is offset by changes in physical activity. Indeed, individuals may choose to exercise more to allow them to consume more unhealthy foods, and this may differ by education. Or similarly, higher educated individuals may be more aware of the importance of physical activity, but sustaining a higher level of physical activity requires more foods.

I therefore next explore whether education causally affects individuals’ physical activity. For this, I use the Active People Survey (APS) from 2012 to 14; a crosssectional survey of adults in England designed to measure individuals’ levels of physical activity. The survey reports individuals’ demographic characteristics, and asks them about their physical activity over the 4 weeks prior to interview, including participation in over 350 types of sports. Individuals are asked to indicate the number of days in the last 4 weeks they engage in each sport, as well as the usual length of time. In addition, they are asked separately about the amount of walking and cycling they did in the last 4 weeks, which includes anything even if it is only 10 minutes. I derive the total length of time in each activity by taking the product of the number of days and the usual length of time. I then take the sum of all activities and divide this by the number of days in the month to obtain a measure of the total *daily* minutes of physical activity.

As in the LCFS, I only include individuals born between 1934 and 1982 and I do not observe individuals’ month of birth. However, the APS does include the year of birth as well as the month of interview, which allows me to construct lower and upper bounds of the year–month of birth for each individual.^13^ I then drop those where it is unclear whether the individual is born before or after 1 September 1957. As the APS only include individuals’ highest qualification, and not the age at which individuals left school, I create a binary measure of whether the individual has done their O-levels (an exam usually taken in the year the individual turns 16). The first stage results are consistent with the literature that specifies having O-levels as the variable of interest when exploiting the compulsory schooling reform (see e.g. Dickson, Gregg and Robinson (2016)).^14^ The final estimation sample includes 168,516 individuals.

The IV results are presented in Table 7 with the reduced form results in Table C.2 and Figure C.5. The latter show that being born after the compulsory schooling reform increased physical activity by approximately 3.2 minutes per day. Scaling this up to the IV estimates, shown in Table 7, suggests that having O-levels increases the amount of time spent in physical activity by just over an hour a day (63 minutes), compared to not having O-levels. This is equally split between time spent in sports (25 minutes a day), and time spent walking (30 minutes a day), although the latter is no longer significantly different from zero when using the ‘*tF*’ procedure. There is no effect on the time spent cycling. Hence, this suggests that more education, in addition to worsening the quality of dietary purchases, also increases the amount of time individuals spend doing physical activity.^15^ Appendix B shows that these estimates are robust to a set of sensitivity analyses, including restricting the sample to ages with common support in treated and control groups, reducing the bandwidth, and controlling for spouse’s education.

One important issue with the above analysis is that it only reflects the amount of physical activity from sports, walking and cycling, while there may have been further changes in physical activity due to occupational choice. Indeed, the additional education achieved by those born after the reform may have affected the *type* of job individuals select into. The higher educated, on average, have less physically demanding jobs, so the increased time spent walking and doing sports among the higher educated may be to compensate for the reduced physical activity at work. Unfortunately, the APS does not include information on occupational choice or job-related physical activity.

To explore this in more detail, I use the English Longitudinal Study of Ageing (ELSA), a nationally representative dataset focusing on the dynamics of health, social, well-being and economic circumstances of those aged 50+ in England. ELSA reports participants’ occupation using the Standard Occupational Classification 2000 (SOC2000) sub-major groups. To investigate whether the those affected by the reform undertake systematically different amounts of physical activity at work, I assign metabolic equivalents of tasks (METs) to each of the SOC2000 two-digit groups. METs measure the metabolic rate of a particular activity relative to the resting metabolic rate (i.e. the rate when resting, lying down or sitting quietly). For example, a MET of two implies that that activity is twice as strenuous as resting. I assign a MET to all SOC2000 codes in the following way. First, I use the METs assigned to each ISCO-08 code^16^ from Deyaert *et al*. (2017), and convert the ISCO-08 codes to SOC2010 using the mapping from the ONS (2016). Second, I map the SOC2010 to SOC2000 and take the average MET across minor groupings to obtain METs for all two-digit groups, giving me an estimated MET for each ELSA participant based on their SOC2000.^17^

As in the APS, I construct lower and upper bounds of the year–month of birth for each individual. Similar to the LCFS, I use a dummy variable indicating whether the individual left school at age ≥16 as the variable of interest. The OLS estimates show that staying in school until 16 is associated with a significant reduction in METs of 0.42 units. In other words, the higher educated are less physically active at work compared to the lower educated. Estimating the first stage IV specification, I find almost identical results to those in Table 3: those born after the reform are 13 percentage points more likely to stay in school until age 16. The first stage F statistic is 28.7, and the final estimation sample includes 7,946 individuals.^18^

The IV results are presented in Table 8 with the reduced form results in Table C.3 and Figure C.6, Appendix C. Both analyses show no differences in METs between those born before vs. after the compulsory schooling reform. Indeed, the IV estimates in Table 8, suggest that those who stay in school until age 16 have slightly higher METs compared to those who leave school before age 16, but with a large standard error, this is not statistically significantly different from zero.

### Back-of-the-envelope calculation

The above estimates suggest that education increases the number of calories purchased by approximately 620 kcal per person per day, while simultaneously increasing the amount of physical activity by about 1 hour per person per day, driven by an additional half hour of walking and half hour of doing sports, with no significant changes in job-related METs. To get an idea of the extent of potential offsetting behaviours, I would ideally translate the changes in physical activity into estimates for calories out. However, this is not straightforward, since the measures of education differ between the two datasets used for the analyses. Indeed, the dietary composition analyses estimates the effect of leaving school at 16 or older, whereas the analyses on physical activity estimates the effect of obtaining O-levels. To ensure that the exposure of interest is identical across the different models, I next present some back-of-the envelope calculations based on the reduced form models, reconciling the estimates.

Table C.1 and Table C.2 present the reduced form parameters, showing that being born in or after 1958 increases calorie purchases by approximately 73 kcal per day (this is equivalent to e.g. half a packet of crisps, or 1.5 Oreo cookies), with an increase in physical activity of 3.2 minutes per day (or 1.8 minutes walking and 1.4 minutes of sports).^19^ Running the reduced form analysis by gender, I find that men, on average, increase their physical activity by 4.8 minutes per day (2.5 minutes walking and 2.4 minutes of sports). For women, the increase is 2.2 minutes per day (1.4 minutes walking and 0.8 minutes of sports). I can use this to create gender-specific estimates of calories *out* that are driven by the increase in physical activity. I do not create gender-specific estimates of calories *in*, since the data on nutrients are not individual-specific (but rather at the household level; I only estimate an increase in individual energy purchase by scaling it with the ‘nutrient equivalence scale’).

First, assuming that the average man in the data weighs 189 lbs^20^ and walks with a speed of 4mph, they are likely to burn an additional 14 kcal in the extra 2.5 minutes spent walking (relative to resting that time instead). Women burn an additional 7 kcal in the 1.4 minutes spent walking. For the additional 2.4 and 0.8 minutes of sports for men and women respectively, the amount of energy burnt depends how strenuous this exercise is. For light physical activity (e.g. playing with children), the average man and woman burn an additional 11 and 3 kcal respectively (again, relative to resting). For moderate physical activity (e.g. baseball, cricket, golf), it is an additional 17 kcal for men and 5 for women, and for vigorous physical activity (e.g. running), it translates to an additional 31 and 9 kcal for men and women respectively.^21^

Combining these estimates suggests that the minimum increase in the amount of energy expended due to the additional time in physical activity (i.e. with light physical activity) is 25 kcal (=14+11) for men and 10 kcal (7+3) for women. The maximum (i.e. with vigorous physical activity) is 30 kcal (=14+31) for men and 16 kcal (7+9) for women. It is important to note that these are very crude estimates for at least two reasons. First, weight is endogenous to both nutrient purchases and physical activity. And second, the estimates of the amount of energy burned in different activities are only an approximation. Having said that, however, they do suggest that there are some partially offsetting effects in terms of calorie intake and expenditure. Note also that the increase in calories *in* is likely to be over-estimated due to higher educated individuals expected to purchase more expensive or higher quality foods within the same food group. The back-of-the-envelope calculation therefore suggests that at least some of the increase in calories *in* is mitigated by an increase in calories *out* through physical activity, leading to little effect on the balance of calories; consistent with the literature that explores the effect of education on obesity (see Galama *et al*., 2018).

### VI Conclusion and discussion

This paper explores the effects of education on dietary choices, as well as potential compensatory responses in terms of physical activity. To account for the endogeneity of education, I exploit the 1972 UK schooling reform that increased the age at which individuals were legally allowed to leave full-time education from 15 to 16 for those born after September 1957, but not for those born before. The results, perhaps surprisingly, show that increased education *worsens* the overall quality of the diet, causing increases in the purchases of calories, fats and animal protein. These results are robust across a wide set of model specifications, including the use of an alternative data source, controlling for a proxy for income, restricting the sample to ages with common support in treated and control groups, using different bandwidths, and controlling for spouse’s level of education (these are shown in Appendix B). However, although the estimates for sugar, non-milk extrinsic sugars, starch and saturated fats are all positive, they are not always precisely estimated, and the results for sugar and nonmilk extrinsic sugar are less robust across different specifications in terms of the size of the coefficients.

How do these estimates compare to the literature? Are there other interventions that lead to similar-sized changes in nutritional purchases? This is difficult to say, since much of the literature explores interventions that aim to reduce BMI (rather than those that specifically focus on nutritional intake, see e.g. Cawley *et al*., 2019), examines changes in quantities consumed (e.g. millilitres per day of sugar-sweetened beverages, see Rayman *et al*., 2018, or kilograms of fruit and vegetables purchased, see Griffith, O’Connell and Smith, 2018), or focuses on a particular mealtime/shopping occasion, often without data on individuals’ consumption or purchases at other times/elsewhere (see e.g. Bollinger, Leslie and Sorensen, 2011; Downs and Loewenstein, 2012). There are, however, some interesting comparisons. For example, Belot *et al*. (2018) find some evidence of changes in children’s (but not adults’) nutritional choices (and BMIs) following two randomised interventions that provided healthy meals or snacks to low-income families. In another randomized experiment, Wisdom, Downs and Loewenstein (2010) show that making healthy choices more convenient in a fast-food chain led to a reduction in calorie purchases, although this is compensated by increased calories on side orders and drinks, and Fichera and von Hinke (2020) and Dubois *et al*. (2021) find that nutrition labelling led to a small improvement in the dietary composition of the shopping basket.

In none of these studies, however, is the ‘treatment effect’ of similar magnitude to what I find here. Indeed, the estimates in this paper remain relatively large in comparison, but there are at least three explanations for this. First, a systematic correlation between education and nutritional quality *within* food groups may lead to an overestimate of the effect of interest in the case of assortative mating. Second, the omission of spouse’s education may lead to an upward bias in the estimates. And third, the IV results should be interpreted as a local average treatment effect. Hence, the estimates should be interpreted as an upper bound of the ‘true’ effect.

Most of the literature that looks at the health effects of additional education either finds improvements in health, or no changes. Finding that education reduces the nutritional quality of the diet is therefore somewhat unexpected and calls for an investigation into the possible mechanisms. I explore multiple potential explanations. First, education may cause a worsening of the diet through an income effect. More specifically, it is well known that the additional schooling due to the reform increased individuals’ wages (see e.g. Harmon and Walker (1995); Oreopoulos (2006)). With more disposable income, these individuals may purchase more foods, increasing the total amount of energy and nutrients purchased. I explore this channel by controlling for total household spending as a proxy for income. The estimates from these analyses remain very similar, suggesting that the increased income associated with the educational reform is not driving my results.

Second, I investigate the role of alcohol and smoking in the relationship between education and nutrition. Indeed, alcohol is often considered a complement to food, while smoking may be a substitute. Increased alcohol intake may therefore coincide with increased food purchases. The analysis shows some suggestion of education increasing spending on alcohol, but not tobacco, which may explain some of the increased nutrient purchases among the group who stayed in school until age 16.

Third, although the higher educated may increase their food *purchases*, they may not necessarily increase their food *consumption* to the same extent. For example, there may be more wastage among higher educated households, or individuals may be more likely to purchase foods for others (e.g. presents or entertaining). I explore this directly in Appendix B by using data from the National Diet and Nutrition Survey, the only source of nationally representative UK data on nutritional *intakes*. The analyses, however, show similar patterns of results, but with substantially smaller samples, the findings are not significantly different from zero.

A fourth potential explanation for the worsening of the diet is that consumers may compensate for healthy food choices using unhealthy ones. For example, Trivedi, Sridhar and Kumar (2016) find that when individuals purchase healthy foods, they may simultaneously buy a more palatable (but less healthy) food. Hence, if the higher educated spend more on healthy foods, such compensatory behaviours may also increase spending on unhealthy foods, including those high in energy and fat.

Finally, I investigate other behavioural effects that may potentially offset the deterioration in the diet. In particular, I explore the effects of education on physical activity. Indeed, individuals may choose to exercise more to allow them to consume more unhealthy foods (i.e. a licensing effect), and this may differ by education. Or similarly, higher educated individuals may be more aware of the importance of physical activity (i.e. a knowledge effect), but a higher level of physical activity requires more food intake. Results confirm a discontinuity in physical activity by birth cohort, with those who got their O-levels because of the compulsory schooling reform being significantly more physically active per day. Although the analysis on physical activity clearly shows the importance of exploring potential offsetting behaviours, I am unable to identify the exact mechanism (e.g. a licensing vs. knowledge effect) that is driving the deterioration of the diet.

A back-of-the-envelope calculation suggests that the increase in calories *in* is only slightly larger than the increase in calories *out*. Together with the specific measurement error in calories in, this suggests there is little effect on the actual calorie balance. This is consistent with the literature that finds no convincing evidence that education causes changes in obesity. Although I am unable to say which comes first (i.e. do the higher educated eat more because they do more exercise, or do they do more exercise because they eat more?), the analysis highlights the importance of separately exploring the effects on calories in vs. calories out, providing additional information that is concealed in the analyses that only use measures of obesity. As such, this study sheds further light on the behavioural responses to an increase in the years of schooling.

One important question relates to what the estimates of education are capturing. First, it is well known that the UK schooling reform led to higher wages for those affected, potentially leading to an increase in nutrients purchased due to higher disposable incomes. However, I show that the estimates are robust to controlling for a proxy for income. Second, the schooling reform may have led to differential selection into job *types* and changes in employment more generally (see e.g. del Bono and Galindo-Rueda, 2004), with accompanying changes in individuals’ time constraints. With the higher educated on average having less strenuous jobs, the reform may have caused a reduction in job-related physical activity, potentially offset by additional discretionary exercise outside of work. I explore this by investigating whether those affected by the reform had different occupational METs, but I find no evidence to support this hypothesis.

Nevertheless, the results in this paper highlight the importance of separately estimating the effects of more disaggregated behaviours, especially when these can be seen as potentially offsetting in their impacts on outcomes such as obesity. Indeed, this paper shows that ignoring the separate subcomponents and only considering the final outcome of interest may conceal different compensating effects.

## Supplementary Material

Web appendix

## Figures and Tables

**Table 1:** Descriptive statistics

	1934-1982	
	Mean	SD
Male	0.336	(0.473)
Age	47.992	(12.172)
Married	0.639	(0.480)
Single	0.158	(0.365)
% of total spend (main shopper)	0.829	(0.177)
No. of adults: 2	0.558	(0.497)
No. of adults: 3+	0.152	(0.359)
No. of children: 1	0.148	(0.355)
No. of children: 2+	0.198	(0.399)
North East	0.043	(0.204)
North West	0.115	(0.319)
Yorkshire	0.083	(0.277)
East midlands	0.073	(0.260)
West midlands	0.085	(0.279)
East	0.092	(0.289)
London	0.122	(0.327)
South East	0.134	(0.341)
South West	0.086	(0.281)
Wales	0.049	(0.215)
Scotland	0.089	(0.285)
Northern Ireland	0.028	(0.166)
N	55581	

*Notes*: Descriptives of the LCFS (2003–15), including individuals with a year of birth between 1934–82. All individual-level characteristics refer to the main shopper in the household.

**Table 2:** OLS results

	(1) Nutient profile score	(2) Calories (kcal)	(3) Carbs (g)	(4) Total sugar (g)	(5) NMES (g)	(6) Starch (g)	(7) Fibre (g)	(8) Fats (g)	(9) Saturated fats (g)	(10) Sodium (g)	(11) Proteins (g)	(12) Vegetable proteins (g)	(13) Animal proteins (g)
Left school ≥16	−0.25 (0.02) [−0.3,−0.2]	−50.76 (16.92)[−84.8,−16.7]	−9.01 (1.79) [−12.6,−5.4]	−1.57 (1.01) [−3.6,0.5]	−5.24 (0.88) [−7.0,−3.5]	−13.87 (2.86) [−19.6,−8.1]	0.68 (0.13) [0.4,0.9]	−3.20 (0.83) [−4.9,−1.5]	−0.97 (0.29) [−1.5,−0.4]	−0.13 (0.02) [−0.2,−0.1]	−0.21 (0.54) [−1.3,0.9]	1.12 (0.24) [0.6,1.6]	−1.32 (0.37) [−2.1, −0.6]
Mean	2.0	2609.7	304.5	134.0	89.1	385.0	20.2	103.0	37.8	3.2	84.6	37.9	46.7
Observations	55581	55581	55581	55581	55581	55581	55581	55581	55581	55581	55581	55581	55581

*Notes:* The table gives the relationship between education and nutritional purchases for a sample of individuals bom between 1934 and 1982. All regressions are estimated using pooled waves of the Living Cost and Food Survey 2003–15. All estimates include a quadratic in the year of birth, a quadratic in age, year and month dummies, gender, marital status, variables indicating the number of adults and the number of children of age 0, age 1, ..., age 17, and region dummies. The confidence intervals are the standard 95% confidence intervals. ‘Mean’ is the mean of the dependent variable (i.e. the average NPS or daily household nutrient purchases) for cohorts bom in the 2 years prior to the policy change. NMES stands for non-milk extrinsic sugars. Robust standard errors clustered by cohort in parentheses.

**Table 3:** First stage IV results

	Pr(Left school ≥16)			
	(1)	(2)	(3)	(4)
I[YOB≥1958]	0.16 (0.02) [0.13,0.19]	0.12 (0.02) [0.09,0.15]	0.12 (0.02) [0.08,0.15]	0.12 (0.02) [0.09,0.15]
1 ^*st*^ stage F-stat	95.1	56.0	55.0	53.3
f(YOB)	Yes	Yes	Yes	Yes
D × f(YOB)	No	Yes	Yes	Yes
Age, age^2^	No	No	Yes	Yes
Covariates	No	No	No	Yes
Outcome mean	0.73	0.73	0.73	0.73
No. of observations	55581	55581	55581	55581

*Notes*: The table shows the estimated effect of the 1972 compulsory schooling law change on the probability of leaving school at age ≥16 in the LCFS (2003-15), including individuals born between 1934 and 1982. The covariates include those mentioned in the note to Table 2. The confidence intervals are the standard 95% confidence intervals. The ‘Outcome mean’ is the mean of the dependent variable for cohorts born in the 2 years prior to the school leaving reform. Robust standard errors clustered by cohort in parentheses.

**Table 4:** Second stage IV results

	(1) Nutrient profile score	(2) Calories (kcal)	(3) Carbs (g)	(4) Total sugar (g)	(5) NMES (g)	(6) Starch (g)	(7) Fibre (g)	(8) Fats (g)	(9) Saturated fats (g)	(10) Sodium (g)	(11) Proteins (g)	(12) Vegetable proteins (g)	(13) Animal proteins (g)
Left school ≥16	0.50 (0-45) [−0.5,1-5]	618.93 (249.58) [77.3,1160.5]	49.84 (27.91) [−10.7,110.4]	34.44 (16.86) [−2.1,71.0]	33.48 (15.66) [−0.5,67.5]	98.87 (54.82) [−20.1,217.8]	−2.91 (2.57) [−8.5,2.7]	32.16 (14.45) [0.8,63.5]	10.52 (5.40) [−1.2,22.2]	0.48 (0.32) [−0.2, 1.2]	11.12 (7.46)[−5.1,27.3]	−5.91 (5.47) [−17.8,6.0]	17.02 (6.56) [2.8,31.3]
Mean	2.0	2609.7	304.5	134.0	89.1	385.0	20.2	103.0	37.8	3.2	84.6	37.9	46.7
Observations	55581	55581	55581	55581	55581	55581	55581	55581	55581	55581	55581	55581	55581

*Notes*: The table gives the IV estimates of education on nutritional purchases for a sample of individuals bom between 1934 and 1982, where education is instrumented using the 1972 compulsory schooling law. All regressions are estimated using pooled waves of the Living Cost and Food Survey 2003–15. The covariates include those mentioned in the note to Table 2. Confidence intervals are calculated using the ‘*tF*” test procedure from Lee *et al*. (2020). ‘Mean’ is the mean of the dependent variable (i.e. the average daily household nutrient purchases) for cohorts bom in the 2 years prior to the policy change. NMES stands for Non-Milk Extrinsic Sugars. Robust standard errors clustered by cohort in parentheses.

**Table 5:** Controlling for total spending

	(1) Nutrient profile score	(2) Calories (kcal)	(3) Carbs (g)	(4) Total sugar (g)	(5) NMES (g)	(6) Starch (g)	(7) Fibre (g)	(8) Fats (g)	(9) Saturated fats (g)	(10) Sodium (g)	(11) Proteins (g)	(12) Vegetable proteins (g)	(13) Animal proteins (g)
Panel A: Linear Left school ≥16	0.47 (0.43) [−0.5,1-4]	642.71 (251.00) [98.0,1187.4]	51.46 (27.70) [−8.6,111.6]	35.34 (16.87) [−1.3,71.9]	33.77 (15.56) [−0.0,67.5]	100.24 (54.04) [−17.0,217.5]	−2.63 (2.50) [−8.1,2.8]	33.04 (14.47) [1.6,64.4]	10.91 (5.41) [−0.8,22.7]	0.51 (0.32) [−0.2,1.2]	12.27 (7.58) [−4.2,28.7]	−5.10 (5.32) [−16.7,6.5]	*17.37* (6.58) [3.1,31.7]
Panel B: Quadratic Left school ≥16	0.55 (0.44) [−0.4,1-5]	574.58 (262.28) [5.4, 1143.7]	45.66 (28.30) [−15.8, 107.1]	32.62 (17.87) [−6.2,71.4]	32.79 (16.28) [−2.5,68.1]	93.64 (54.66) [−25.0,212.2]	−3.45 (2.44) [−8.7,1.8]	30.83 (15.18) [−2.1,63.8]	9.97 (5.65) [−2.3,22.2]	0.42 (0.34) [−0.3,1.1]	9.08 (8.02) [−8.3,26.5]	−7.28 (5.06) [−18.3,3.7]	16.36 (7.03) [1.1,31.6]
Mean	2.0	2609.7	304.5	134.0	89.1	385.0	20.2	103.0	37.8	3.2	84.6	37.9	46.7
Observations	55581	55581	55581	55581	55581	55581	55581	55581	55581	55581	55581	55581	55581

*Notes:* See notes to Table 4. Confidence intervals are calculated using the ‘*tF*’ test procedure from Lee *et al*. (2020). Panel A additionally controls for a linear specification of total spending (as a proxy for income); Panel B includes a quadratic in total spending. NMES stands for Non-Milk Extrinsic Sugars.

**Table 6:** Second stage IV results, alcohol and tobacco spending

	(1) Any spending on alcohol (0/1)	(2) Alcohol spending (£)	(3) Any spending on tobacco (0/1)	(4) Tobacco spending (£)
Left school ≥16	0.24 (0.12) [−0.0,0.5]	1.63 (0.80) [−0.1,3.4]	−0.06 (0.09) [−0.3,0.1]	0.45 (0.40) [−0.4,1.3]
Mean	0.7	2.4	0.3	0.7
Observations	55581	55581	55581	55581

*Notes:* The table gives the IV estimates of education on purchases of / spending on alcohol and tobacco for a sample of individuals born between 1934 and 1982, where education is instrumented using the 1972 compulsory schooling law. All regressions are estimated using pooled waves of the Living Cost and Food Survey 2003-15. The covariates include those mentioned in the note to Table 2. Confidence intervals are calculated using the *‘tF’* test procedure from Lee *et al*. (2020). ‘Mean’ is the mean of the dependent variable for cohorts born in the 2 years prior to the policy change. Robust standard errors clustered by cohort in parentheses.

**Table 7:** Second stage IV results, Active People Survey

	(1) Total daily physical activity (in minutes)	(2) Total daily PA (less walking) (in minutes)	(3) Total daily walking (in minutes)	(4) Total daily cycling (in minutes)
O-levels	63.22 (19.74) [22.7,103.8]	24.70 (7.04) [10.2,39.2]	29.65 (15.89) [−3.0,62.3]	1.14 (2.74) [−4.5,6.8]
Mean	45.7	10.3	33.1	2.2
Observations	168516	168516	168516	168516

*Notes*: The table gives the IV estimates for the effect of education on physical activity (in minutes) for a sample of individuals born between 1934 and 1982. All regressions are estimated using pooled waves of the Active People Survey 2012-14. All estimates include a quadratic in the date of birth and that interacted with the treatment dummy, a quadratic in age, year and month dummies, gender, and variables indicating the number of adults and children in the household. Confidence intervals are calculated using the ‘*tF*’ test procedure from Lee *et al*. (2020). ‘Mean’ is the mean of the dependent variable for cohorts born in the 2 years prior to the policy change. Robust standard errors clustered by cohort in parentheses.

**Table 8:** Second stage IV results, english longitudinal study of ageing

	(1) MET
Left school at age ≥16	0.07 (0.34) [−0.7,0.9]
Mean	2.2
Observations	7946

*Notes*: The table gives the IV estimates for the effect of education on metbolic equivalents of tasks (METs) for a sample of individuals born between 1934 and 1982. All regressions are estimated using one observation per person from the English Longitudinal Study of Ageing. All estimates include a quadratic in the year of birth and that interacted with the treatment dummy, a quadratic in age, year and month dummies, gender, marital status and region dummies. Confidence intervals are calculated using the ‘*tF*’ test procedure from Lee *et al*. (2020). ‘Mean’ is the mean of the dependent variable for cohorts born in the 2 years prior to the policy change. Robust standard errors clustered by cohort in parentheses.

## Footnotes

1 Other indirect effects could run through education affecting individuals’ time preferences, making them more future oriented (Fuchs (1982)), which in turn can lead to better health outcomes and behaviours. Similarly, higher educated individuals may respond differently to stress or anxiety (e.g. comfort eating vs. exercising), or have systematically different peers and therefore be exposed to different behaviours and habits.

2 Other approaches that have been used to explore the effect of education include randomized controlled trials and twin studies (for a review of this literature, see Galama *et al*., 2018).

3 These are high-quality data, being used by the UK Government to define the ‘basket of goods’ for the consumer price index (CPI) and the retail price index (RPI), the former of which is used to uprate benefits, tax credits and public service pensions. The data are also used by HM Treasury and HM Revenues and Customs to study how taxes and benefits affect household income and to study the effects of policy in these areas (ONS, 2012).

4 This ‘nutrient equivalence scale’ takes into account the recommended daily energy intakes by age and gender, based on the Committee on Medical Aspects of Food Policy (COMA), published by the Department of Health (1991). More specifically, for each individual in the household, I take their (age and gender specific) recommended energy intake as a proportion of the adult equivalent (i.e. 1900 kcal for 50-59-year-old women, and 2550 kcal for 50-59-year-old men) and sum this up over all household members. I use this to scale the total household nutrient purchases to individual purchases. However, the results are robust to using other equivalence scales, such as the OECD equivalence scales, the OECD modified equivalence scales, not using any equivalence scales, or simply dividing the amount of nutrients by the total number of household members.

5 These are also known as ‘added sugars’ or ‘free sugars’, and include only those added to food and drinks.

6 This cut-off ensures that individuals in the sample were not differentially affected by the 1947 compulsory schooling reform, as this affected those born from 1 April 1933 onwards.

7 Distinguishing between household size, this is 100% for single-person households, 81% for two-person households, 76% for three-person households and 75% for four or more person households.

8 I use a quadratic polynomial approach, rather than the global high-order polynomial approximation discussed in Gelman and Imbens (2019)

9 As the different dependent variables sum up to the total household nutritional purchases, the errors are likely to be correlated across equations for a given household. In principle, this cross-equation correlation of the errors can be exploited to improve the efficiency of the estimates, using a three-stage least squares (3SLS) procedure. However, as the equations are just-identified, the 3SLS reduces to a 2SLS estimation, presented below.

10 Table C.1, Figure C.3 and Figure C.4, Appendix C, show the reduced form effects. Graphs showing the mean of the *covariates* by year of birth show no evidence of discontinuities, suggesting that the individuals observed on either side of the cut-point are similar in observable characteristics (available upon request).

11 For example, evidence shows that some individuals smoke at least in part to control their weight (Cawley, Dragone and von Hinke, 2014), and that smoking causally reduces body weight (van Kippersluis and Rietveld, 2018). There is evidence that the higher educated are more likely to drink alcohol, despite alcohol-related deaths being concentrated among lower SES groups (see e.g. Collins, 2016).

12 The OLS estimates show that education is positively correlated with alcohol spending, but negatively correlated with spending on tobacco. Both are significantly different from zero at conventional levels. These are not shown here, but are available upon request.

13 For example, a 50-year-old individual who is interviewed in January 2012 was born between January 1962 and February 1963.

14 The results show that the reform increased the probability of having O-levels by five percentage points relative to the pre-reform mean of 19%, with a first stage F-statistic of 69. Results are available upon request.

15 This finding is consistent with Buckles *et al*. (2013) who find that college graduates are more likely to exercise compared to non-college graduates.

16 The International Standard Classification of Occupations (ISCO-08) classifies jobs into 436 groups.

17 There is no 1:1 mapping between SOC2000 and SOC2010, with one of the main revisions in SOC2010 being the reclassifications of managerial functions. I have incorporated these based on the redefinitions provided in (Elias and Birch, 2010). Hence, although there is generally good overlap between the sub-major groupings of SOC2000 and SOC2010 (see e.g. Elias and Birch, 2010), there is likely to remain some incorrect classifications leading to measurement error.

18 Note that although ELSA is a panel, I only include one observation per individual, since education and occupation are mostly constant within individuals over time, especially at older ages.

19 To ensure the estimates for calories in and out are based on a similar sample, I drop the year 1957 in both analyses and control for quadratic annual trends in the year of birth. Including the year 1957, identifying those born before and after September 1957, and specifying year-month trends for the analyses on the APS provides very similar results, which are available upon request.

20 Or 86kg. This is the average weight among men born in 1957/8 and observed between 2003 and 12 in the Health Survey for England (own calculations). The amount of energy burnt is a function of an individual’s body weight.

21 These numbers are taken from statistics provided by the Harvard Medical School (https://www.health.harvard.edu/diet-and-weight-loss/calories-burned-in-30-minutes-of-leisure-and-routine-activities). For example, a 185 lbs individual burns 26 kcal when resting for 30 minutes (or 0.9 kcal per minute). The same individual burns 189 kcal in 30 minutes walking (6.3 kcal p/m). Hence, spending an additional 2.5 minutes walking implies burning between 16 kcal *minus* 2 kcal (i.e. what would be burnt if the individual had rested this time instead), so approximately 14 kcal.
