# Supplementary material for: Education, Dietary Intakes and Exercise
Source: Oxf Bull Econ Stat. Author manuscript; Available in PMC 2023 Dec 7. (PMC7615357; doi:10.1111/obes.12463)
Supplement: Web appendix [file EMS191865-supplement-Web_appendix.pdf]

Education, dietary intakes and exercise

— Online Appendix —

Stephanie von Hinke*

* School of Economics, University of Bristol, Priory Road Complex, Priory Road, Bristol, BS8 1TU; Erasmus School of Economics, Erasmus University Rotterdam; and Institute for Fiscal Studies, S.vonHinke@bristol.ac.uk.

Appendix A: Nutritional Profile Model

To measure the nutritional quality, I use the Nutritional Profiling Model that was developed by the UK FSA (Rayner et al. (2009)). This model scores both 'positive' (e.g. fibre) and 'negative' (e.g. sugar) nutrients, and incorporates these into a score indicating the 'healthiness' of the diet.

The nutritional quality is calculated by summing up the number of 'negative' nutrient points, allocated based of the nutritional content per 100g of the shopping basket, and then subtracting the number of 'positive' nutrient points, allocated in a similar way, to obtain a total score. Points are allocated for each of the following individual nutrients: energy, saturated fats, total sugars, sodium, % fruit, vegetables and nuts, fibre and protein. The tables below shows the points for each nutrient, depending on the content per 100g.

Table A.1: Points for 'positive' and 'negative' nutrients

	Points for 'negative' nutrients				Points for 'positive' nutrients		
Points	Energy (kJ)	Saturated fats (g)	Total sugar (g)	Sodium (mg)	Fruit, veg & nuts (%)	Fibre (g)	Protein (g)
0	≤335	≤1	≤4.5	≤90	≤40	≤0.9	≤1.6
1	>335	>1	>4.5	>90	>40	>0.9	>1.6
2	>670	>2	>9	>180	>60	>1.9	>3.2
3	>1005	>3	>13.5	>270	-	>2.8	>4.8
4	>1340	>4	>18	>360	-	>3.7	>6.4
5	>1675	>5	>22.5	>450	>80	>4.7	>8.0
6	>2010	>6	>27	>540			
7	>2345	>7	>31	>630			
8	>2680	>8	>36	>720			
9	>3015	>9	>40	>810			
10	>3350	>10	>45	>900			

To calculate the total score, the model subtracts the 'positive' points from the 'negative' points if the household scores less than 11 'negative' points in total. If the household scores 11 or more 'negative' points, the total score is computed by subtracting the fibre points from the 'negative' score. The FSA defines a food product as 'less healthy' if it scores 4 points or more; a drink is defined as 'less healthy' if it scores 1 point or more.

The original FSA model also subtracts the 'positive' from the 'negative' score if the total 'negative' score is 11 or more with a score of 5 for fruit, vegetables and nuts. The LCSF data do not allow me to distinguish between the fruit, vegetable and nut content.

Appendix B: Robustness analyses

This appendix explores the sensitivity of the IV estimates for the analyses on nutritional choices as well as (if data allow) physical activity. I start by investigating whether the negative effect on dietary quality can be replicated in a different dataset. I then examine the robustness of the findings with respect to (i) restricting the sample to ages with common support in treated and control groups, (ii) the bandwidth in the RD analyses, and (iii) the inclusion of the spouse's level of education. I also examine (iv) whether the effect of education is similar for *food in* versus *food out*, and (v) I investigate any heterogeneity, exploring whether the effect of education on dietary choices is similar for men and women, and those with and without children.

B.1 National Diet and Nutrition Survey

To explore whether the reduction in diet quality is consistent across different datasets, I start by exploring the National Diet and Nutrition Survey (NDNS): a cross-sectional survey recording the types and quantities of foods *consumed* (as opposed to *purchased*) by individuals. These data are available for six years: from 2008/09 to 2013/14. This survey, however, is substantially smaller than the LCFS, with approximately 500 individuals (aged 19+) observed each year. The survey reports individuals' demographic characteristics and asks them to keep a four-day diary recording all food and drinks consumed each day. I use the average (per person) daily consumption of energy and nutrients as the outcomes of interest.

I only include individuals born between 1934 and 1982. Similar to the APS, as year-month of birth is not recorded in the data, I use individuals' year of birth and month of interview to construct lower and upper bounds of the year-month of birth for each individual. I drop those where it is unclear whether the individual is born before or after 1 September 1957.

The first stage IV estimates are almost identical to those using the LCFS: being born in or after September 1957 increases the probability of leaving school at age $\geq$16 by 11 percentage points, from a baseline of 73%. However, with a much smaller sample size (approximately 6.5% of that in the LCFS), the first stage F-statistic is 7.2 (the estimates are not shown here, but are available upon request). The second stage IV estimates are presented in Table B.1, confirming the earlier findings that additional education increases the consumption of energy, carbohydrates, sugars, fibre and protein. However, with the small sample size and large standard errors, none of the estimates are significantly different from zero. Nevertheless, this suggests that the general pattern of results observed for *food purchases* in the LCFS replicates to *food consumption* in the NDNS.

B.2 Common support

The main sample for the analyses on nutritional choices includes all birth cohorts from 1934 to 1982 who are observed in the LCFS between 2003 and 2015, therefore covering a relatively large age range. Although the analyses control for a quadratic in age, it may be that some of the estimates reflect age effects, as opposed to those driven by the schooling reform. I therefore

next restrict the sample to only include ages with common support in both 'treated' (i.e. born in/after 1958) and 'control' (born before 1958) groups in all LCFS waves. In other words, in the 2003 wave, the youngest treated cohort is 45 years old (i.e. born in 1958), with older individuals born before the schooling reform and therefore in the control group. Similarly, in the 2015 wave, the oldest control cohort is 58 years old (i.e. born in 1957), with younger individuals born after the schooling reform and therefore in the treated group. Table B.2 presents the estimates from analysis that restricts the sample to those aged 45 to 58 at the time they were observed. This shows similar estimates, albeit slightly larger. The estimates now also show an increase in purchases of carbohydrates, starch, fibre, sodium, and both vegetable and animal proteins.

B.3 Bandwidth

Identification of the effect of education relies on the treatment being as good as randomly assigned near the cut-point. The smaller the bandwidth, the more likely that this holds. However, reducing the bandwidth also reduces the power, making it more difficult to identify the effects of interest. The main model specification includes 24 birth cohorts on either side of the cut-point. To explore the robustness of the results, Table B.3, Table B.4, and Table B.5 present the findings using a bandwidth of 20 years (Panel A), 15 years (Panel B), 10 years (Panel C), and 5 years (Panel D) for nutritional choices, sports-related and occupational physical activity respectively. Table B.3 shows relatively robust estimates for calories, carbohydrates, fats, saturated fats, and proteins. For example, the estimates show that education increases the number of calories purchased by between 682 and 898 kcal. The results that are less robust though, are sugar and NMES, where the estimates for larger bandwidths show that additional education increases sugar purchases, but this turns negative when the bandwidth is specified at five years (though both are insignificant). This also affects the overall Nutritional Profile Score, which is turns negative with bandwidth of 5 or 10 years.

Table B.4 and Table B.5 shows the results for daily physical activity and METs respectively. The magnitude of the estimates is similar to those in Table 7 and Table 8. For physical activity, however, reducing the bandwidth renders the estimates insignificantly different from zero. For example, using a 5-year bandwidth shows that having O-levels increases the amount of time spent in physical activity by just over 50 minutes a day; equally split between time spent in sports (25 minutes) and time spent walking (22 minutes). There is again no effect on the time spent cycling. The estimates for METs are slightly more variable, but are insignificant in all specifications.

I cannot do this analysis for the APS, since individuals are only interviewed between 2012-2014, restricting the age range with common support to 54–57, leading to a very small sample. However, this common support issue becomes less of a problem when restricting the bandwidth, the results of which I present below. The findings show that, although the estimates are no longer significant due to a lack of power, the effect sizes are very similar in magnitude.

B.4 Accounting for spouse's education

The education reform may have led to complex general equilibrium effects. For example, if it caused an increase in marriages *within* cohorts due to assortative mating, the coefficient on the main shopper's education may partially capture the effect of the spouse's education. The main analysis above ignores the latter, since spouse's education is missing for many observations. Indeed, I do not observe spouse's education for 34% of the estimation sample, leading to a large drop in the sample size. However, omitting this may lead to an upward bias in the coefficient estimates. I therefore explore the robustness of my analysis here, including spouse's education in the estimation.

Similar to the main analysis, I use a dummy variable indicating whether the spouse left school at age 16 or later and I instrument this using a dummy for whether the spouse was born in or after 1958, controlling for a quadratic in their year of birth and age. I additionally drop observations where the spouse is born in 1957 to avoid erroneously assigning individuals to being born before or after the threshold. The estimates are presented in Table B.6. As expected, this shows smaller (though still large) estimates. However, with the substantially larger standard errors and the 'tF' correction, none of the estimates are significantly different from zero.

B.5 Eating in or out

Table B.7 investigates whether education matters more for nutritional purchases *in the home* (Panel A) versus *out of the home* (Panel B). Before interpreting the estimates, it is useful to note that the average household daily nutrient purchases vary substantially between purchases in and outside the home. This is shown in the row indicated "mean" in both panels. With an average nutrient profile score of 1.4 for food in compared to 13 for food out, foods eaten outside the home are substantially *less* healthy. Furthermore, just under one tenth of all energy and nutrients that are purchased by households, are for outside consumption (e.g. 222 kcal for food out versus 2387 kcal for food in).

The OLS estimates of the effect of education show that the higher educated eat more healthily inside the home, but their choices are less healthy away from home. After instrumenting for education, however, it shows that additional education leads to an increase in purchases of energy and nutrients, both inside and outside of the home, though they are not always significant using the inflated 'tF' confidence intervals. For example, those who left school at age 16 because of the reform increased their purchases of energy (kcal) by 545 kcal inside the home, and by 73 kcal outside the home, though the latter is not significant. This suggests that additional education worsens dietary choices more generally, rather than being restricted to either food in or out.

B.6 Heterogeneous effects

Table B.8 and Table B.9 explore whether the effect of education differs by subgroups of the population. Panels A and B of Table B.8 present the estimates for female and male shoppers respectively, showing that additional education increases purchases of calories, NMES, starch, fats, saturated fats, sodium and animal proteins for both genders. However, with the reduced sample size and adjusted confidence intervals, most of these are not significantly different from zero. Any significant effects, however, are only for female shoppers. Table B.9 shows that education increases physical activity for both men and women, but the largest effects are found for men. Indeed the effect sizes for men are double those for women, suggesting that the increase in physical activity for cohorts born after the schooling reform are driven by males.

Panels C and D present the results for households with and without children respectively. Table B.8 shows that the worsening of the diet are driven by the latter, with the results for households with children being generally smaller in magnitude (or negative). However, with the reduced sample size and more conservative confidence intervals, they are generally not significantly different from zero. Although the estimates for physical activity in Table B.9 are larger for families with children, they are not statistically significantly different from zero. Instead, the estimates for families without children suggest they increase their physical activity by 52 minutes per day; 19 of which are due to increased exercise and 24 minutes due to increased walking.

Table B.1: Second stage IV results, National Diet and Nutrition Survey

	(1) Energy (kcal)	(2) Carbohydrates (g)	(3) Total sugar (g)	(4) NMES (g)	(5) Starch (g)	(6) Fibre (g)	(7) Fats (g)	(8) Sats (g)	(9) Proteins (g)
Left school at age ≥16	463.84	100.69	70.31	21.37	30.66	2.15	-0.36	-4.63	22.25
	(434.84)	(72.17)	(44.77)	(31.07)	(39.41)	(5.34)	(19.96)	(9.24)	(22.69)
	[-656.2,1583.9]	[-85.2,286.6]	[-45.0,185.6]	[-58.7,101.4]	[-70.9,132.2]	[-11.6,15.9]	[-51.8,51.0]	[-28.4,19.2]	[-36.2,80.7]
Mean	1712.5	213.6	90.8	50.8	122.7	14.1	69.1	26.1	72.6
Observations	3668	3668	3668	3668	3668	3668	3668	3668	3668

Notes: The table gives the IV estimates of education on nutritional intakes. All regressions are estimated using pooled waves of the National Diet and Nutrition Survey 2008/09-2013/14, and include a quadratic in the year of birth and that interacted with the treatment dummy, a quadratic polynomial in age, year and month dummies, gender, marital status, household size, and regional dummies. Confidence intervals are calculated using the 'tF' test procedure from Lee et al. (2020). NMES stands for Non-Milk Extrinsic Sugars. Robust standard errors clustered by birth year-month in parentheses.

Table B.2: Robustness analyses: Common support, Living Cost and Food Survey

	(1) Nutrient Profile Score	(2) Calories (kcal)	(3) Carbs (g)	(4) Total sugar (g)	(5) NMES (g)	(6) Starch (g)	(7) Fibre (g)	(8) Fats (g)	(9) Saturated fats (g)	(10) Sodium (g)	(11) Proteins (g)	(12) Vegetable proteins (g)	(13) Animal proteins (g)
Left school ≥16	-0.71	813.86	84.80	24.58	15.79	214.41	5.52	29.16	9.50	0.94	33.19	14.29	18.90
	(0.58)	(272.05)	(26.27)	(19.54)	(16.39)	(39.88)	(2.47)	(14.78)	(4.99)	(0.37)	(9.84)	(4.15)	(7.60)
	[-2.0,0.5]	[223.5,1404.2]	[27.8,141.8]	[-17.8,67.0]	[-19.8,51.4]	[127.9,300.9]	[0.2,10.9]	[-2.9,61.2]	[-1.3,20.3]	[0.1,1.7]	[11.8,54.5]	[5.3,23.3]	[2.4,35.4]
Mean	2.0	2615.6	305.2	133.9	89.1	389.0	20.3	103.2	37.8	3.2	84.7	38.1	46.7
Observations	17814	17814	17814	17814	17814	17814	17814	17814	17814	17814	17814	17814	17814

Notes: Restricting the sample to ages 45–58. Confidence intervals are calculated using the 'tF' test procedure from Lee et al. (2020). See notes to Table 4. Confidence intervals are calculated using the 'tF' test procedure from Lee et al. (2020). NMES stands for Non-Milk Extrinsic Sugars.

Table B.3: Robustness analyses: Varying the bandwidth, Living Cost and Food Survey

	(1) Nutrient Profile Score	(2) Calories (kcal)	(3) Carbs (g)	(4) Total sugar (g)	(5) NMES (g)	(6) Starch (g)	(7) Fibre (g)	(8) Fats (g)	(9) Saturated fats (g)	(10) Sodium (g)	(11) Proteins (g)	(12) Vegetable proteins (g)	(13) Animal proteins (g)
**Panel A: 20y**													
Left school ≥16	0.37	785.42	77.10	43.26	38.25	150.01	-0.47	37.15	12.00	0.64	15.12	0.34	14.78
	(0.47)	(291.04)	(30.53)	(21.58)	(19.12)	(51.12)	(2.34)	(16.47)	(6.17)	(0.34)	(8.39)	(4.28)	(7.59)
	[-0.7,1.4]	[153.8,1417.0]	[10.8,143.3]	[-3.6,90.1]	[-3.2,79.7]	[39.1,261.0]	[-5.5,4.6]	[1.4,72.9]	[-1.4,25.4]	[-0.1,1.4]	[-3.1,33.3]	[-8.9,9.6]	[-1.7,31.3]
No. of observations	49697	49697	49697	49697	49697	49697	49697	49697	49697	49697	49697	49697	49697
**Panel B: 15y**													
Left school ≥16	0.02	682.12	66.32	19.35	18.83	200.33	1.06	35.72	10.03	0.39	13.14	3.84	9.31
	(0.46)	(299.19)	(29.95)	(17.07)	(15.52)	(45.95)	(2.40)	(17.54)	(6.08)	(0.32)	(8.57)	(3.93)	(6.72)
	[-1.0,1.0]	[32.8,1331.4]	[1.3,131.3]	[-17.7,56.4]	[-14.8,52.5]	[100.6,300.0]	[-4.1,6.3]	[-2.3,73.8]	[-3.2,23.2]	[-0.3,1.1]	[-5.5,31.7]	[-4.7,12.4]	[-5.3,23.9]
No. of observations	41301	41301	41301	41301	41301	41301	41301	41301	41301	41301	41301	41301	41301
**Panel C: 10y**													
Left school ≥16	-0.18	898.41	93.92	24.61	25.55	235.61	3.39	34.90	15.05	1.14	35.91	11.96	23.95
	(0.53)	(255.04)	(25.66)	(17.16)	(18.12)	(43.81)	(2.40)	(13.95)	(5.54)	(0.33)	(10.51)	(3.75)	(9.43)
	[-1.3,1.0]	[345.0,1451.9]	[38.2,149.6]	[-12.6,61.9]	[-13.8,64.9]	[140.5,330.7]	[-1.8,8.6]	[4.6,65.2]	[3.0,27.1]	[0.4,1.9]	[13.1,58.7]	[3.8,20.1]	[3.5,44.4]
No. of observations	28611	28611	28611	28611	28611	28611	28611	28611	28611	28611	28611	28611	28611
**Panel D: 5y**													
Left school ≥16	-0.50	830.42	117.49	-13.53	-27.06	237.38	7.86	30.03	19.71	0.23	16.24	15.86	0.38
	(0.87)	(621.08)	(90.51)	(37.57)	(31.85)	(163.06)	(4.94)	(34.78)	(15.75)	(0.76)	(15.25)	(11.63)	(10.83)
	[-2.4,1.4]	[-517.4,2178.2]	[-78.9,313.9]	[-95.1,68.0]	[-96.2,42.1]	[-116.5,591.2]	[-2.9,18.6]	[-45.5,105.5]	[-14.5,53.9]	[-1.4,1.9]	[-16.9,49.3]	[-9.4,41.1]	[-23.1,23.9]
No. of observations	13617	13617	13617	13617	13617	13617	13617	13617	13617	13617	13617	13617	13617

Notes: See notes to Table 4. Confidence intervals are calculated using the 'tF' test procedure from Lee et al. (2020). NMES stands for Non-Milk Extrinsic Sugars.

Table B.4: Robustness analyses: Varying the bandwidth, Active People Survey

	(1) Total daily physical activity (in minutes)	(2) Total daily PA (less walking) (in minutes)	(3) Total daily walking (in minutes)	(4) Total daily cycling (in minutes)
**Panel A: 20y bandwidth**				
O-levels	52.84	29.06	11.58	1.67
	(30.87)	(11.31)	(25.21)	(4.41)
	[-10.6,116.2]	[5.8,52.3]	[-40.2,63.4]	[-7.4,10.7]
Observations	149557	149557	149557	149557
**Panel B: 15y bandwidth**				
O-levels	38.30	16.32	0.32	2.55
	(36.47)	(12.42)	(30.15)	(5.45)
	[-36.6,113.2]	[-9.2,41.8]	[-61.6,62.2]	[-8.6,13.7]
Observations	119286	119286	119286	119286
**Panel C: 10y bandwidth**				
O-levels	38.25	4.17	25.39	3.66
	(34.05)	(10.76)	(27.73)	(4.82)
	[-31.7,108.2]	[-17.9,26.3]	[-31.6,82.3]	[-6.2,13.6]
Observations	79972	79972	79972	79972
**Panel D: 5y bandwidth**				
O-levels	52.32	25.06	21.52	0.19
	(80.68)	(34.16)	(60.29)	(11.76)
	[-113.4,218.0]	[-45.1,95.2]	[-102.3,145.3]	[-24.0,24.3]
Observations	39254	39254	39254	39254

Notes: See notes to Table 7. Confidence intervals are calculated using the 'tF' test procedure from Lee et al. (2020).

Table B.5: Robustness analyses: Varying the bandwidth, English Longitudinal Study of Ageing

	(1)<br>MET
**Panel A: Bandwidth of 20 years**	
Left school at age ≥16	-0.12
	(0.33)
	[-0.9,0.7]
Observations	7247
**Panel B: Bandwidth of 15 years**	
Left school at age ≥16	-0.24
	(0.38)
	[-1.1,0.6]
Observations	6125
**Panel C: Bandwidth of 10 years**	
Left school at age ≥16	-0.44
	(0.30)
	[-1.1,0.3]
Observations	4497
**Panel D: Bandwidth of 5 years**	
Left school at age ≥16	0.43
	(0.58)
	[-0.9,1.8]
Observations	2336

Notes: See notes to Table 4. Confidence intervals are calculated using the 'tF' test procedure from Lee et al. (2020).

Table B.6: Robustness analyses: Accounting for spouse's edcuation

	(1) Nutrient Profile Score	(2) Calories (kcal)	(3) Carbs (g)	(4) Total sugar (g)	(5) NMES (g)	(6) Starch (g)	(7) Fibre (g)	(8) Fats (g)	(9) Saturated fats (g)	(10) Sodium (g)	(11) Proteins (g)	(12) Vegetable proteins (g)	(13) Animal proteins (g)
Left school ≥16	0.19	458.35	40.32	32.72	33.55	119.12	-2.48	22.49	7.74	0.20	9.13	-7.90	17.03
	(0.52)	(307.71)	(36.30)	(18.62)	(16.56)	(104.44)	(2.92)	(15.57)	(5.60)	(0.38)	(8.89)	(6.06)	(8.34)
	[-0.9,1.3]	[-209.4,1126.1]	[-38.5,119.1]	[-7.7,73.1]	[-2.4,69.5]	[-107.5,345.8]	[-8.8,3.8]	[-11.3,56.3]	[-4.4,19.9]	[-0.6,1.0]	[-10.2,28.4]	[-21.0,5.2]	[-1.1,35.1]
Mean	2.0	2608.9	304.6	134.2	89.3	380.4	20.1	103.0	37.8	3.2	84.4	37.7	46.7
Observations	36469	36469	36469	36469	36469	36469	36469	36469	36469	36469	36469	36469	36469

Notes: See notes to Table 4. The analysis also controls for spouse's education (a dummy whether they left school at age ≥16), instrumented by a dummy indicating whether they are born in or after 1958, whilst controlling for a quadratic in the spouse's year of birth and age. IV confidence intervals are calculated using the 'tF' test procedure from Lee et al. (2020).

Table B.7: Robustness analyses: Eating in or eating out, Living Cost and Food Survey

	(1) Nutient Profile Score	(2) Calories (kcal)	(3) Carbs (g)	(4) Total sugar (g)	(5) NMES (g)	(6) Starch (g)	(7) Fibre (g)	(8) Fats (g)	(9) Saturated fats (g)	(10) Sodium (g)	(11) Proteins (g)	(12) Vegetable proteins (g)	(13) Animal proteins (g)
**Pabel A: Eating in**													
<u>OLS:</u> Left school ≥16	-0.13	-89.32	-13.05	-2.49	-5.74	-10.31	0.40	-5.10	-1.54	-0.19	-1.83	-0.51	-1.32
	(0.03)	(15.90)	(1.71)	(0.98)	(0.85)	(1.00)	(0.12)	(0.80)	(0.28)	(0.02)	(0.52)	(0.20)	(0.37)
	[-0.2,-0.1]	[-121.3,-57.3]	[-16.5,-9.6]	[-4.5,-0.5]	[-7.4,-4.0]	[-12.3,-8.3]	[0.2,0.6]	[-6.7,-3.5]	[-2.1,-1.0]	[-0.2,-0.1]	[-2.9,-0.8]	[-0.9,-0.1]	[-2.1,-0.6]
<u>IV:</u> Left school ≥16	0.23	545.82	43.07	31.56	30.74	11.79	-3.06	30.33	9.90	0.46	9.63	-7.39	17.02
	(0.53)	(241.06)	(27.14)	(16.13)	(14.61)	(18.89)	(2.37)	(14.45)	(5.31)	(0.31)	(7.53)	(4.54)	(6.56)
	[-0.9,1.4]	[22.7,1068.9]	[-15.8,102.0]	[-3.4,66.6]	[-1.0,62.4]	[-29.2,52.8]	[-8.2,2.1]	[-1.0,61.7]	[-1.6,21.4]	[-0.2,1.1]	[-6.7,26.0]	[-17.2,2.5]	[2.8,31.3]
Mean	1.4	2387.3	282.8	124.9	81.6	154.2	19.0	94.3	35.1	2.9	77.4	30.7	46.7
Observations	55581	55581	55581	55581	55581	55581	55581	55581	55581	55581	55581	55581	55581
**Pabel B: Eating out**													
<u>OLS:</u> Left school ≥16	1.26	38.57	4.04	0.93	0.50	3.04	0.28	1.89	0.57	0.06	1.62		
	(0.11)	(2.98)	(0.27)	(0.13)	(0.12)	(0.16)	(0.02)	(0.12)	(0.03)	(0.00)	(0.09)		
	[1.0,1.5]	[32.6,44.6]	[3.5,4.6]	[0.7,1.2]	[0.3,0.7]	[2.7,3.4]	[0.2,0.3]	[1.7,2.1]	[0.5,0.6]	[0.1,0.1]	[1.4,1.8]		
<u>IV:</u> Left school ≥16	7.12	73.11	6.77	2.88	2.74	3.94	0.15	1.84	0.62	0.02	1.48		
	(1.53)	(36.15)	(3.24)	(1.86)	(1.84)	(3.19)	(0.37)	(1.94)	(0.56)	(0.05)	(1.67)		
	[3.8,10.4]	[-5.3,151.6]	[-0.3,13.8]	[-1.2,6.9]	[-1.3,6.7]	[-3.0,10.9]	[-0.6,0.9]	[-2.4,6.0]	[-0.6,1.8]	[-0.1,0.1]	[-2.1,5.1]		
Mean	13.0	222.4	21.7	9.1	7.5	12.3	1.1	8.7	2.7	0.3	7.1		
Observations	55581	55581	55581	55581	55581	55581	55581	55581	55581	55581	55581		

Notes: See notes to Table 4. OLS confidence intervals are calculated with a 'standard' critical value of 1.96. IV confidence intervals are calculated using the 'tF' test procedure from Lee et al. (2020). Data on the purchases of animal proteins are not available for eating out, so the table only reports purchases of vegetable proteins in Panel B.

Table B.8: Robustness analyses: Heterogeneous effects by subgroups, Living Cost and Food Survey

	(1) Nutrient Profile Score	(2) Calories (kcal)	(3) Carbs (g)	(4) Total sugar (g)	(5) NMES (g)	(6) Starch (g)	(7) Fibre (g)	(8) Fats (g)	(9) Saturated fats (g)	(10) Sodium (g)	(11) Proteins (g)	(12) Vegetable proteins (g)	(13) Animal proteins (g)
**A: Female**													
Left school ≥16	0.44	487.35	66.97	46.31	38.47	102.45	-1.72	15.55	9.16	0.58	11.81	-5.60	17.42
	(0.43)	(253.75)	(27.77)	(19.43)	(17.73)	(60.05)	(2.53)	(13.05)	(5.24)	(0.37)	(8.74)	(6.38)	(5.66)
	[-0.5,1.4]	[-63.3,1038.0]	[6.7,127.2]	[4.1,88.5]	[-0.0,77.0]	[-27.9,232.8]	[-7.2,3.8]	[-12.8,43.9]	[-2.2,20.5]	[-0.2,1.4]	[-7.2,30.8]	[-19.4,8.2]	[5.1,29.7]
No. of observations	38213	38213	38213	38213	38213	38213	38213	38213	38213	38213	38213	38213	38213
**B: Male**													
Left school ≥16	0.72	843.15	-10.07	-3.46	16.68	76.16	-6.92	71.53	12.29	0.08	4.98	-7.36	12.33
	(0.98)	(575.22)	(60.38)	(22.70)	(21.28)	(78.36)	(5.16)	(40.54)	(10.50)	(0.67)	(14.61)	(10.08)	(13.99)
	[-1.4,2.8]	[-405.1,2091.4]	[-141.1,121.0]	[-52.7,45.8]	[-29.5,62.9]	[-93.9,246.2]	[-18.1,4.3]	[-16.4,159.5]	[-10.5,35.1]	[-1.4,1.5]	[-26.7,36.7]	[-29.2,14.5]	[-18.0,42.7]
No. of observations	17368	17368	17368	17368	17368	17368	17368	17368	17368	17368	17368	17368	17368
**C: Children**													
Left school ≥16	0.72	78.66	-29.23	-18.63	-2.61	123.51	-10.94	7.70	2.23	-0.06	-9.45	-14.53	5.08
	(1.04)	(520.16)	(62.79)	(30.26)	(28.74)	(200.57)	(9.77)	(26.83)	(8.55)	(0.63)	(23.03)	(16.70)	(12.43)
	[-1.5,3.0]	[-1050.1,1207.5]	[-165.5,107.0]	[-84.3,47.0]	[-65.0,59.7]	[-311.7,558.8]	[-32.1,10.3]	[-50.5,65.9]	[-16.3,20.8]	[-1.4,1.3]	[-59.4,40.5]	[-50.8,21.7]	[-21.9,32.1]
No. of observations	20049	20049	20049	20049	20049	20049	20049	20049	20049	20049	20049	20049	20049
**D: No children**													
Left school ≥16	0.52	691.22	61.06	37.63	36.28	103.86	-1.78	33.85	10.46	0.57	14.35	-3.28	17.63
	(0.46)	(325.97)	(38.75)	(19.12)	(15.78)	(54.02)	(2.83)	(17.30)	(6.36)	(0.39)	(8.78)	(4.95)	(7.08)
	[-0.5,1.5]	[-16.2,1398.6]	[-23.0,145.1]	[-3.9,79.1]	[2.0,70.5]	[-13.4,221.1]	[-7.9,4.4]	[-3.7,71.4]	[-3.3,24.3]	[-0.3,1.4]	[-4.7,33.4]	[-14.0,7.5]	[2.3,33.0]
No. of observations	35532	35532	35532	35532	35532	35532	35532	35532	35532	35532	35532	35532	35532

Notes: See notes to Table 4. Confidence intervals are calculated using the 'tF' test procedure from Lee et al. (2020).

Table B.9: Robustness analyses: Heterogeneous effects by subgroups, Active People Survey

	(1)<br>Total daily<br>physical activity<br>(in minutes)	(2)<br>Total daily<br>PA (less walking)<br>(in minutes)	(3)<br>Total daily<br>walking<br>(in minutes)	(4)<br>Total daily<br>cycling<br>(in minutes)
**Panel A: Female**				
O-levels	47.40	16.21	26.36	3.07
	(20.47)	(6.61)	(17.44)	(2.37)
	[5.4,89.4]	[2.6,29.8]	[-9.5,62.2]	[-1.8,7.9]
Observations	99698	99698	99698	99698
**Panel B: Male**				
O-levels	100.89	43.03	37.61	-2.84
	(44.57)	(16.36)	(35.26)	(7.13)
	[9.3,192.4]	[9.4,76.6]	[-34.8,110.0]	[-17.5,11.8]
Observations	68818	68818	68818	68818
**Panel C: Children**				
O-levels	87.63	27.28	14.85	15.69
	(62.29)	(23.05)	(47.95)	(9.71)
	[-40.3,215.6]	[-20.1,74.6]	[-83.6,113.3]	[-4.3,35.6]
Observations	45453	45453	45453	45453
**Panel D: No children**				
O-levels	51.81	19.33	24.27	-1.37
	(22.42)	(7.64)	(18.09)	(2.90)
	[5.8,97.9]	[3.6,35.0]	[-12.9,61.4]	[-7.3,4.6]
Observations	123063	123063	123063	123063

Notes: See notes to Table 7. Confidence intervals are calculated using the ‘tF’ test procedure from Lee et al. (2020).

Appendix C: Additional Tables and graphs

Figure C.1: Density of the Nutritional Profile Score

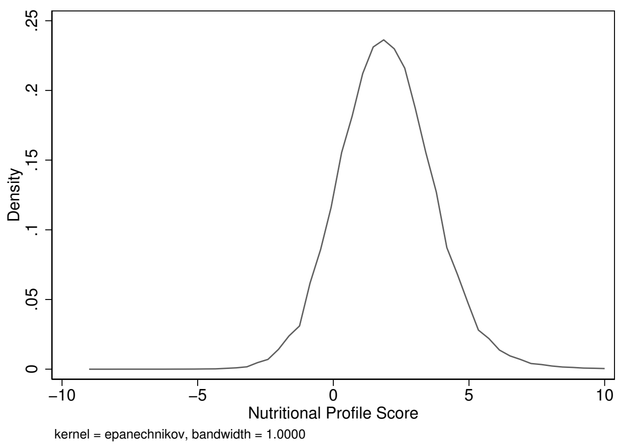

Notes: The figure plots the density of the Nutrient Profile Score from the Living Cost and Food Survey, summarizing the healthiness of households' diets. Each score is based on the total nutrients per 100g of the shopping basket. See Appendix A for more detail.

Figure C.2: Densities of nutrient purchases, 2003-2015

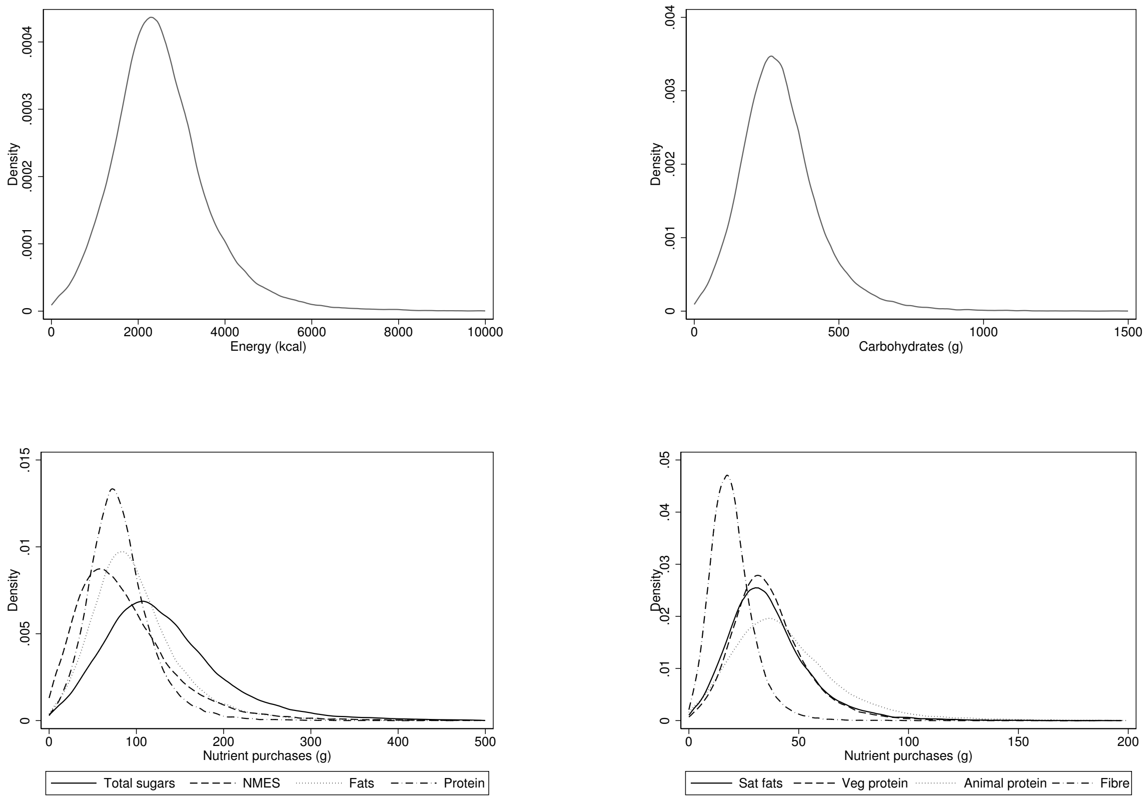

Notes: The figures plot densities of the different nutrients from the Living Cost and Food Survey, where each has been equivalised using the 'nutrient equivalence scale'. Energy is measured in calories (kcal) per person per day, with all other nutrients measured in grams per person per day.

Table C.1: Reduced form results Living Cost and Food Survey

	(1) Nutrient Profile Score	(2) Calories (kcal)	(3) Carbs (g)	(4) Total sugar (g)	(5) NMES (g)	(6) Starch (g)	(7) Fibre (g)	(8) Fats (g)	(9) Saturated fats (g)	(10) Sodium (g)	(11) Proteins (g)	(12) Vegetable proteins (g)	(13) Animal proteins (g)
YOB $\geq$ 1958	0.06	72.89	5.87	4.06	3.94	11.64	-0.34	3.79	1.24	0.06	1.31	-0.70	2.00
	(0.05)	(25.43)	(3.06)	(1.68)	(1.57)	(6.34)	(0.31)	(1.51)	(0.59)	(0.03)	(0.87)	(0.63)	(0.71)
Mean	2.0	2609.7	304.5	134.0	89.1	385.0	20.2	103.0	37.8	3.2	84.6	37.9	46.7
Observations	55581	55581	55581	55581	55581	55581	55581	55581	55581	55581	55581	55581	55581

Notes: The table gives the reduced form estimates for the 1972 compulsory schooling law for a sample of individuals born between 1934 and 1982. All regressions are estimated using pooled waves of the Living Cost and Food Survey 2003-2015. All estimates include a quadratic in the year of birth and that interacted with the treatment dummy, a quadratic in age, year and month dummies, gender, marital status, household size, and region dummies. The confidence intervals are the standard 95% confidence intervals. “Mean” is the mean of the dependent variable for cohorts born in the 2 years prior to the policy change. Robust standard errors clustered by cohort in parentheses.

Figure C.3: The Nutritional Profile Score by year of birth, Living Cost and Food Survey

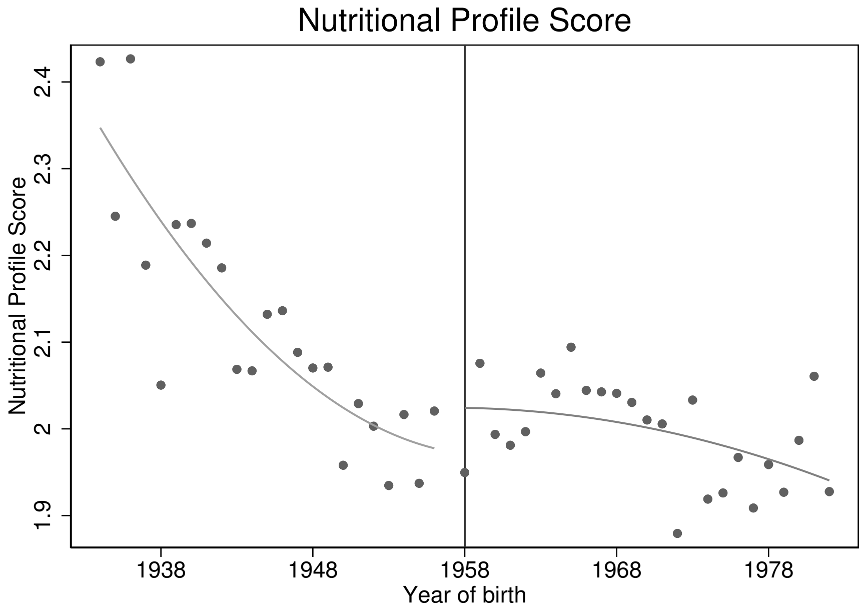

Notes: The figures plot mean values of the Nutient Profile Score on the vertical axis by year of birth on the horizontal axis from the Living Cost and Food Survey, with the vertical line denoting 1958.

Figure C.4: Discontinuities in outcome variables by year of birth, Living Cost and Food Survey

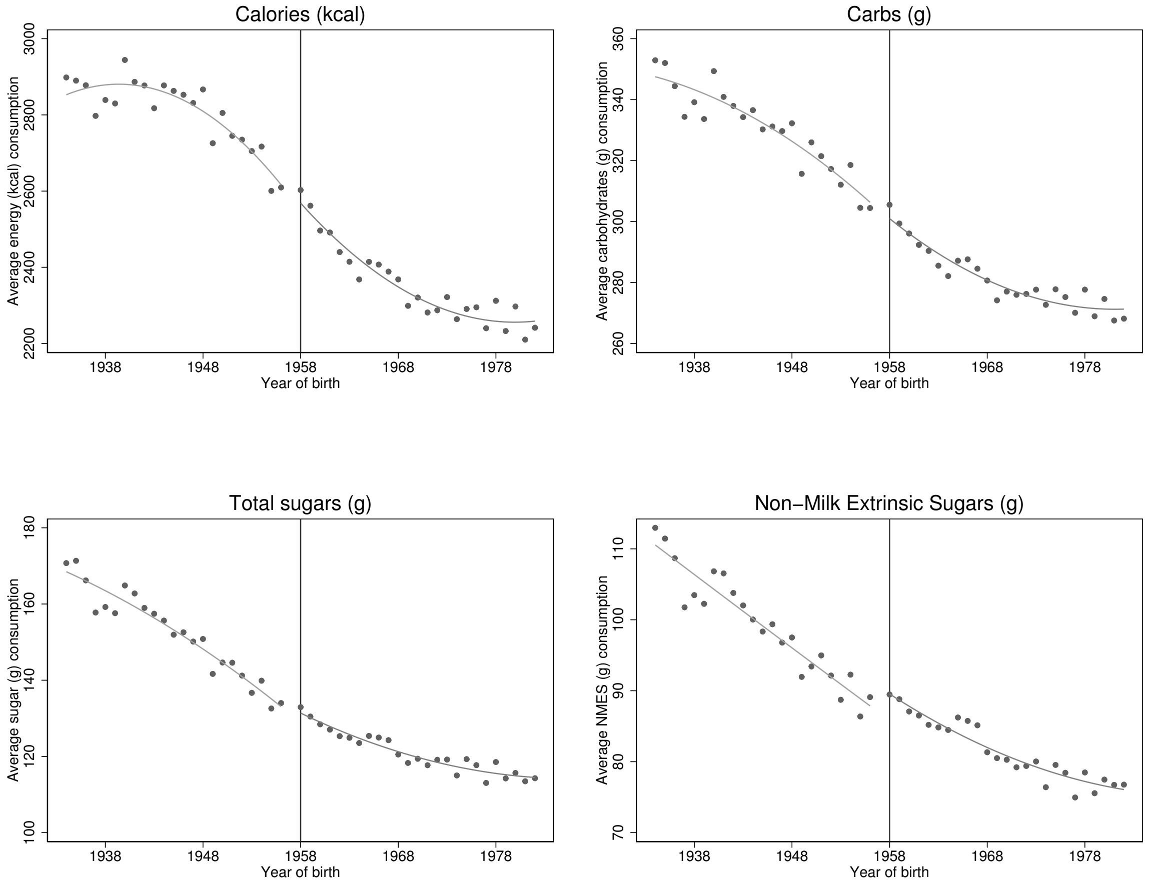

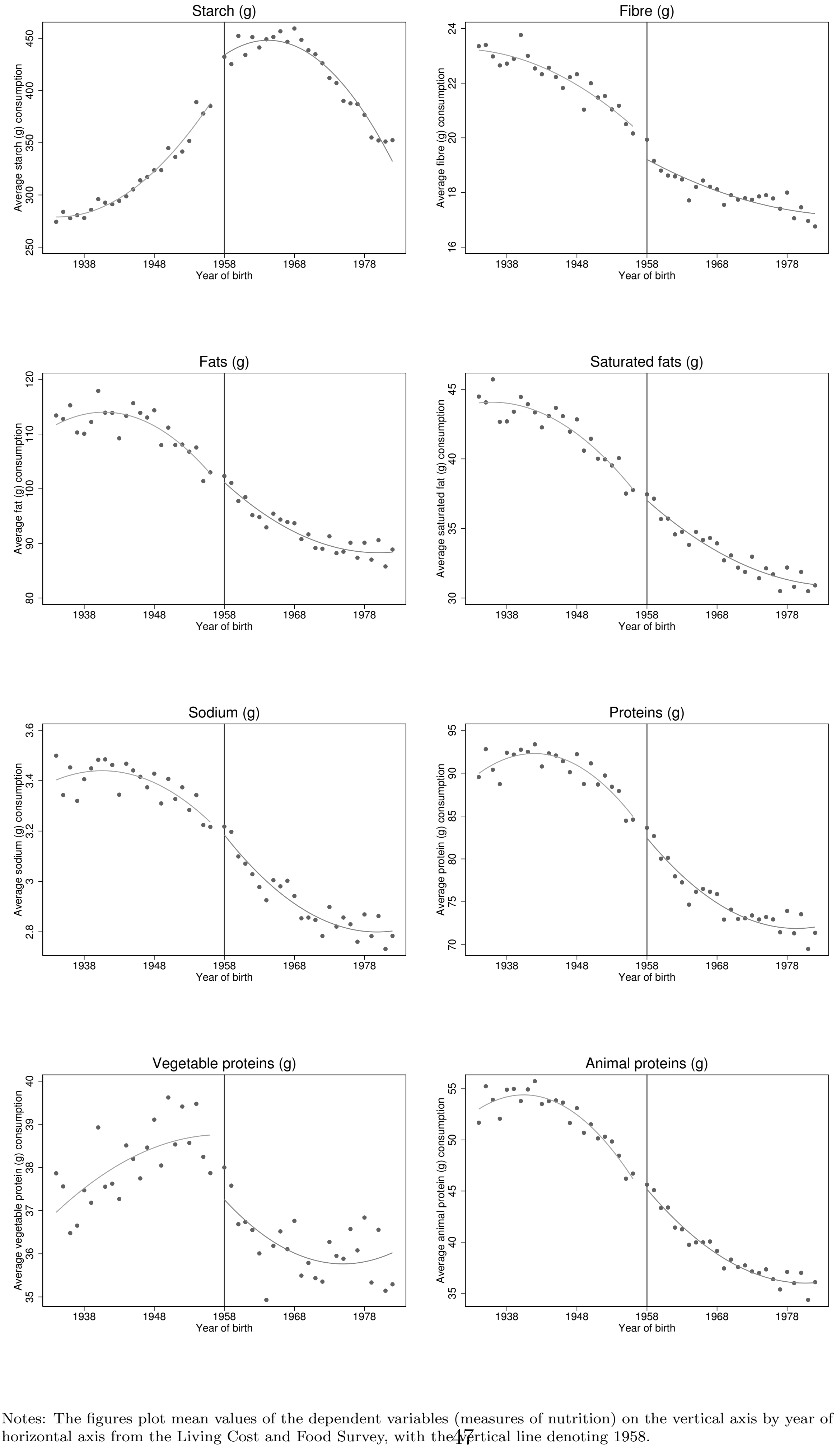

Notes: The figures plot mean values of the dependent variables (measures of nutrition) on the vertical axis by year of birth on the horizontal axis from the Living Cost and Food Survey, with the vertical line denoting 1958.

Table C.2: Reduced form results Active People Survey

	(1) Total daily PA (in minutes)	(2) Total daily PA (less walking) (in minutes)	(3) Total daily walking (in minutes)	(4) Total daily cycling (in minutes)
YOB $\geq$ 1958	3.18	1.44	1.79	-0.06
	(0.82)	(0.39)	(0.62)	(0.12)
Mean				
Observations	328239	328239	328239	328239

Notes: The table gives the reduced form estimates for the 1972 compulsory schooling law for a sample of individuals born between 1934 and 1982. All regressions are estimated using pooled waves of the Active People Survey 2012-2014. All estimates include a quadratic in the year of birth and that interacted with the treatment dummy, a quadratic in age, year and month dummies, gender, and variables indicating the number of adults and children in the household. The confidence intervals are the standard 95% confidence intervals. "Mean" is the mean of the dependent variable for cohorts born in the 2 years prior to the policy change. Robust standard errors clustered by cohort in parentheses.

Figure C.5: Discontinuities in outcome variables by year-month of birth, Active People Survey

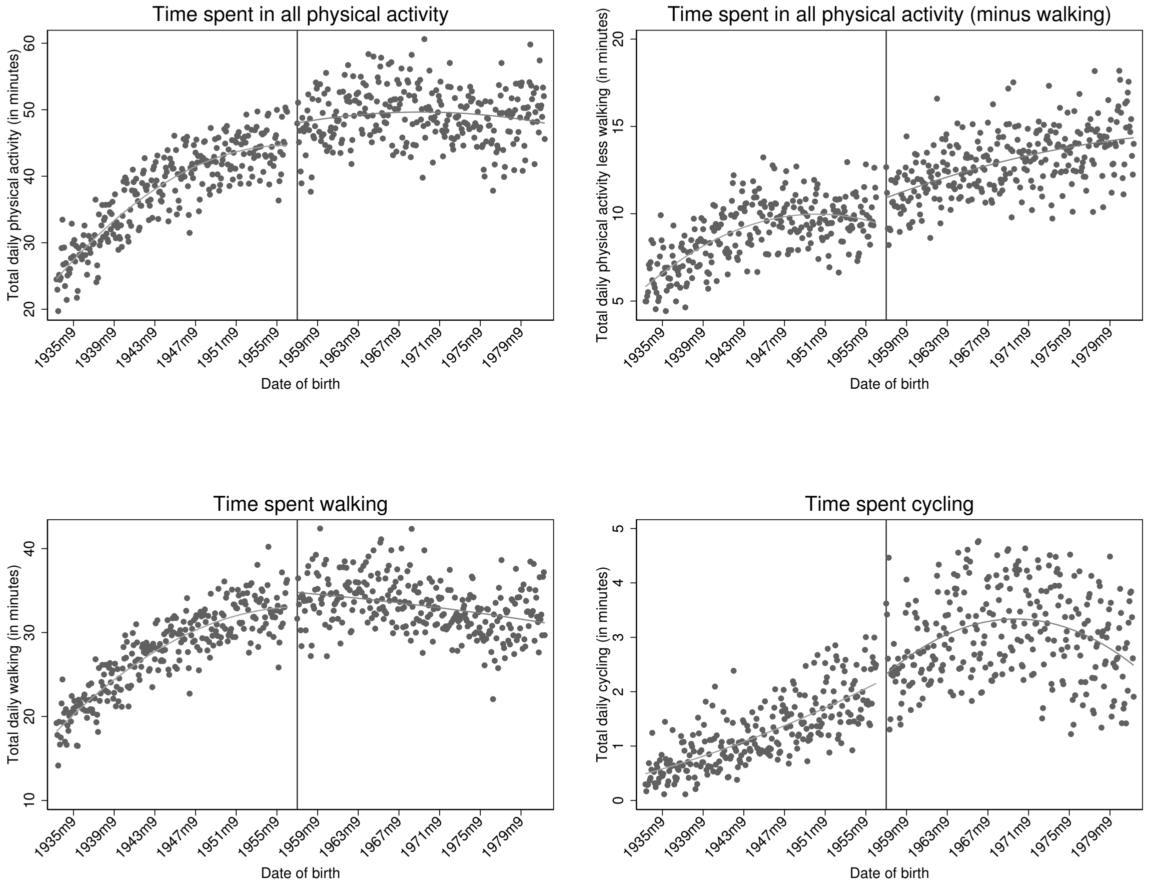

Notes: The figures plot mean values of the dependent variables (measures of physical activity) on the vertical axis by year-month of birth on the horizontal axis from the Active People Survey, with the vertical line denoting September 1957.

Table C.3: Reduced form results English Longitudinal Study of Ageing

	(1) Energy (kcal)
DOB $\geq$ Sept 1957	0.01
	(0.04)
Mean	2.4
Observations	7946

Notes: The table gives the reduced form estimates for the 1972 compulsory schooling law for a sample of individuals born between 1934 and 1982. All regressions are estimated using the English Longitudinal Study of Ageing. All estimates include a quadratic in the year of birth and that interacted with the treatment dummy, a quadratic in age, year and month dummies, gender, marital status and region dummies. The confidence intervals are the standard 95% confidence intervals. "Mean" is the mean of the dependent variable for cohorts born in the 2 years prior to the policy change. Robust standard errors clustered by cohort in parentheses.

Figure C.6: Discontinuities in outcome variables by year-month of birth, English Longitudinal Study of Ageing

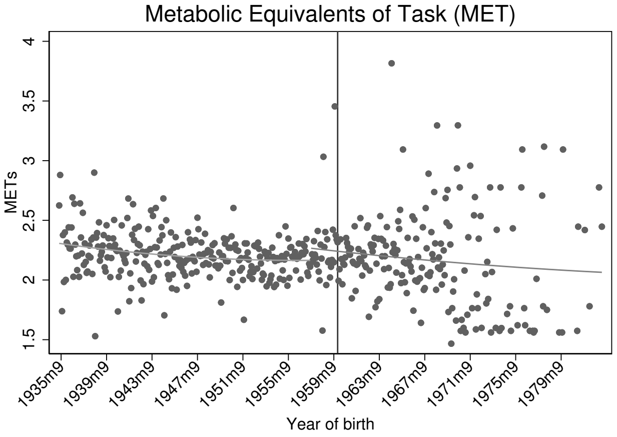

Notes: The figures plot mean values of the dependent variable (Metabolic Equivalents of Task, or METs) on the vertical axis by year-month of birth on the horizontal axis from the English Longitudinal Study of Ageing, with the vertical line denoting September 1957.
